# Interaction of Linear Polyelectrolytes with Proteins: Role of Specific Charge–Charge Interaction and Ionic Strength

**DOI:** 10.3390/biom11091377

**Published:** 2021-09-17

**Authors:** Julia Bukala, Prabhusrinivas Yavvari, Jacek J. Walkowiak, Matthias Ballauff, Marie Weinhart

**Affiliations:** 1 Department of Chemistry and Biochemistry, Freie Universität Berlin, Takustrasse 3, 14109 Berlin, Germany; juliabukala@hotmail.com (J.B.); pyavvari@zedat.fu-berlin.de (P.Y.); jjwalkowiak@zedat.fu-berlin.de (J.J.W.); 2 Institute of Physical Chemistry and Electrochemistry, Leibniz Universität Hannover, Callinstr. 3A, 30167 Hanover, Germany

**Keywords:** polycation, ITC, complex formation, counterion release, thermodynamic analysis

## Abstract

We present a thermodynamic study of the interaction of synthetic, linear polyelectrolytes with bovine serum albumin (BSA). All polyelectrolytes are based on poly(allyl glycidyl ether) which has been modified by polymer-analogous reaction with anionic (-SO_3_Na), cationic (-NH_3_Cl or -NHMe_2_Cl) or zwitterionic groups (-NMe_2_(CH_2_)_3_SO_3_). While the anionic polymer shows a very weak interaction, the zwitterionic polymer exhibits no interaction with BSA (pI = 4.7) under the applied pH = 7.4, ionic strength (I = 23–80 mM) and temperature conditions (T = 20–37 °C). A strong binding, however, was observed for the polycations bearing primary amino or tertiary dimethyl amino groups, which could be analysed in detail by isothermal titration calorimetry (ITC). The analysis was done using an expression which describes the free energy of binding, Δ*G*_b_, as the function of the two decisive variables, temperature, *T*, and salt concentration, *c*_s_. The underlying model splits Δ*G*_b_ into a term related to counterion release and a term related to water release. While the number of released counter ions is similar for both systems, the release of bound water is more important for the primary amine compared to the tertiary *N*,*N*-dimethyl amine presenting polymer. This finding is further traced back to a closer contact of the polymers’ protonated primary amino groups in the complex with oppositely charged moieties of BSA as compared to the bulkier protonated tertiary amine groups. We thus present an investigation that quantifies both driving forces for electrostatic binding, namely counterion release and change of hydration, which contribute to a deeper understanding with direct impact on future advancements in the biomedical field.

## 1. Introduction

Linear polyelectrolytes may form well-defined complexes with proteins in dilute solution. Thus, DNA, RNA, and other natural polyelectrolytes can interact with proteins such as polymerases in aqueous solution, and the complex formation presents a process that has been studied for decades [1]. The obvious biological importance has led to a large number of precise thermodynamic studies [2,3,4,5,6,7,8,9,10,11] that have been reviewed recently [12,13]. Synthetic polyelectrolytes interacting with proteins have also been the subject of intense studies [14,15,16,17,18,19]. The motivation for these investigations is two-fold: On the one hand, polymers are often appended to prevent the adsorption of proteins from aqueous solution [20]. On the other hand, polyelectrolytes may form complex coacervates [16] with proteins that have found various applications, e.g., in food technology [17]. 

**Figure 1:**
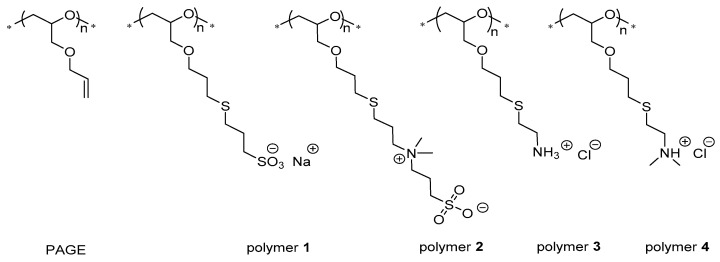
Chemical structure of the precursor poly(allyl glycidyl ether) (PAGE) from which the linear model polyelectrolytes **1**–**4** have been synthesized for investigation in this work (* denotes unspecified polymer end group).

Linear poly(glycidyl ether)s constitute a class of biocompatible polyethers with various applications in the biomedical field covering bioinert [21], antibacterial [22], cell-adhesive [23], as well as switchable [24] polymers and coatings. The functional side groups offer the possibility for further chemical modification and adjustment of the properties of these polyethers to the respective application [25,26]. Concerning cellular adhesion, a universal binding strategy is employed when designing biocompatible polycations which interact mainly electrostatically with the negatively charged cellular glycocalyx [27]. The safe use of such polymers for medical purposes requires a detailed and mechanistic understanding of their interaction with biological systems on all levels. For basic systematic studies, the versatile and efficient conversion of pending allyl groups of poly(allyl glycidyl ether) (PAGE) via thiol-ene chemistry generates a highly comparable set of polymers based on the same polyether backbone with adjustable functionalities [28]. Within the present study, we prepared a series of linear model polyelectrolytes (Figure 1) covering anionic, zwitterionic and cationic pendants to investigate their binding properties with proteins as one of the initial and fate-determining interactions in biological systems. 

At physiological pH, the strong sulfonic acid groups of polymers **1** and **2** are deprotonated, yielding a polyanion and a neutral zwitterion, respectively, while both primary and tertiary amines in polymers **3** and **4** are largely protonated according to their pK_a_ values. Albumin, as one of the most prominent globular proteins in blood plasma and serum (35–50 g/L in humans), carries a net negative charge (pI = 4.7; 66 kDa) and functions as an osmotic pressure stabilizer and endo- and exogenous molecule/ion transporter, as well as an antioxidant in blood [29]. Due to its high capacity for binding and transporting molecules and its omnipresence, both in the intra- and extravascular space, serum albumin serves as a relevant model protein to study polyelectrolyte interactions.

Here we present, as a first step, a thermodynamic study of the interaction of synthetic model polyelectrolytes with bovine serum albumin (BSA). Isothermal titration calorimetry (ITC) is used as the tool for providing a precise determination of the binding constant, *K*_b_ [30]. Previous work has demonstrated that ITC is the method of choice when *K*_b_ has to be obtained with an accuracy high enough to conduct a comprehensive thermodynamic analysis [19,31,32,33]. Recently, it has been shown that binding constant *K*_b_ must be measured as the function of temperature, *T*, and salt concentration, *c*_s_, in order to arrive at an understanding of the main driving forces of binding of proteins to polyelectrolytes [34]: (i) First, complex formation between the protein and the polyelectrolyte will release part of the counterions condensed [35,36,37] to the highly charged polyelectrolyte chain. This counterion release mechanism was established by Record et al. some time ago [1] and has been corroborated in many experimental studies since (cf. the review [12] for further discussion). (ii) The second driving force for binding is the release of water already discussed in the early expositions of the problem [1,38]. A part of the water localized in the hydrate shell of the protein will be released upon binding. Depending on the distribution of the ions between the hydrate and the bulk water, this release will stabilize or destabilize the complex. As shown by Record et al., this effect is intimately related to ion-specific effects embodied in the Hofmeister series [39,40]. 

Both effects have been combined in our recent analysis to yield a closed expression of the free energy of binding, Δ*G*_b_(*T*,*c*_s_), as the function of two decisive variables, temperature and salt concentration [34]. Thus, counterion release leads to a term scaling with ln *c*_s_, while the effect of water release scales linearly with *c*_s_ (see also the discussion of the Hofmeister effects in [39]). The role of temperature, *T*, requires special consideration. The treatment introduced in [34] works in the vicinity of a maximum of the free energy of binding, Δ*G*_b_(*T*,*c*_s_), as the function of *T*. Under these conditions, the entropy of binding is smaller than the specific heat, Δ*c_p_*, which in turn determines the dependence of Δ*G*_b_(*T*,*c*_s_) on temperature. Hence, we shall first analyse the temperature dependence of the free energy of binding in terms of a novel master curve that allows us to compare the results to other systems [33,34]. In a second step, the dependence on *T* will be analysed together with the dependence on *c*_s_. This full analysis [34] of Δ*G*_b_(*T*,*c*_s_) can then be related to the molecular structure of the polyelectrolytes shown in Figure 1.

## 2. Materials and Methods

**Materials.** Allyl glycidyl ether, tetraoctylammonium bromide, 2,2-dimethoxy-2-phenylacetophenone (DMPA) (99%), 2-(dimethylamino)ethanethiol hydrochloride (95%), and sodium 3-mercapto-1-propane sulfonate (90%) were purchased from Sigma–Aldrich (Steinheim, Germany). Cysteamine hydrochloride (97%) from Fluka and triisobutyl aluminum 1.1 M solution in toluene from Acros Organic were purchased from Fisher Scientific (Schwerte, Germany). The betaine precursor 3-((3-mercaptopropyl)dimethylammonio)propane-1-sulfonate was synthesized according to the literature [27]. Allyl glycidyl ether was dried over CaH_2_ and freshly distilled before use in polymerizations. If not stated otherwise, all materials were used without further purification. 

Albumin from bovine serum fraction V (98%) and sodium chloride (≥99%) was received from Carl Roth GmbH+Co. KG (Karlsruhe, Germany). Components of the phosphate buffer sodium phosphate dibasic (≥99%), sodium phosphate monobasic (≥99%) and sodium azide (≥99%) were purchased from Merck Millipore (Darmstadt, Germany). MQ water was prepared via Milli-Q Reference system from Merck with minimum resistivity of 18.2 MΩ·cm and with TOC content < 5 ppb. For dialysis, pre-wetted regenerated cellulose dialysis tubing with a molecular weight cut-off of 3.5 kDa from SpectrumLabs (Fisher Scientific, Schwerte, Germany) was used.

**Synthesis.** Poly(allyl glycidyl ether) (PAGE) was obtained via the monomer activated anionic ring-opening polymerization of allyl glycidyl ether in anhydrous toluene according to the literature [41,42]. In brief, triisobutyl aluminium (1 eq) and tetraoctylammonium bromide (0.25 eq) were employed as activator and initiator, respectively, while the molecular weight was adjusted via the monomer to initiator concentration ratio. The polymerization was performed at 0 °C under argon atmosphere for 3 h at a scale of 40 g monomer, aiming at a molecular weight of 15 kDA. After quenching, the crude polymer was purified via dialysis (MWCO 1 kDa) in toluene and obtained as a viscous oil in 87.5% yield after isolation. GPC analysis indicated a molecular weight of *M*_n_ = 12.1 kDa and a dispersity of *Ð* = 1.19 (Figure S1). 

The functional polymers **1**–**4** were synthesized via UV-initiated thiol-ene post-functionalization of PAGE in the presence of 2,2-dimethoxy-2-phenylacetophenone (DMPA, 0.2 eq) with the respective thiols (sodium 3-mercapto-1-propane sulfonate, cysteamine hydrochloride, 2-(dimethylamino)ethanethiol hydrochloride; 3 eq per allyl group) in methanol or—in the case of polymer **2**—with 3-((3-mercaptopropyl)dimethylammonio)propane-1-sulfonate (3 eq per allyl group) in a mixture of MeOH: trifluoroethanol: water = 1:1:1 at a concentration of ~3 mg PAGE per mL solvent [43]. The photoreactions were performed for 11 h under irradiation with a mercury UV-Vis lamp (70 W; LOT-Oriel, Serial No. 266) at scales ranging from 0.1–2 g. For purification, the crude reaction mixture was first dialyzed against 0.5 M NaCl brine and then excessively against water. After isolation, the product was obtained as a slightly yellow solid in 96, 64, 28, 46% yield for polymers **1**–**4**, respectively. All NMR spectra can be found in the Supplementary Materials (Figures S2–S11).

**Characterization.** Gel permeation chromatography (GPC) of PAGE was performed in tetrahydrofuran at a flow rate of 1 mL min^−1^ at 25 °C, applying polystyrene standards (PSS, Mainz, Germany) on an Agilent 1100 Series instrument (Agilent, Waldbronn, Germany). Three PLgel mixed-C columns (dimensions 7.5 × 300 mm, particle size 5 µm; PSS, Mainz, Germany) were used in-line with a refractive index (RI) detector. The molecular weight of the polyelectrolytes was calculated from the weight-average molecular weight of PAGE and the molecular weight of the respective thiol after confirmation of 100% conversion of all allyl groups via ^1^H NMR. The precursor PAGE and polymers **1**–**4** were characterized by ^1^H-NMR and ^13^C-NMR on a Joel ECX at 400 MHz or a Brucker AVANCE III operating at 700 MHz and 176 MHz, respectively. NMR spectra were processed with the software MestReNova 14.1.1; chemical shifts were referenced to the respective deuterated solvent peak (D_2_O). 

**Isothermal Titration Calorimetry.** ITC experiments were conducted using a Malvern Panalytical (Kassel, Germany) MicroCal VP-ITC instrument, and data was processed with the supplied Microcal module for Origin 7.0 (Additive GmbH, Friedrichsdorf, Germany). All polymer and protein samples were prepared in a phosphate buffer (9 mM; pH 7.4) containing sodium azide (2 mM) to inhibit microbial growth, which overall resulted in a 23 mM ionic strength. Prior the measurement, all samples were degassed and thermostated for 5 min at the temperature of the respective experiment. The BSA solution was titrated into the sample cell loaded with 1.43 mL of the respective polyelectrolyte solution. Polymer and protein concentrations are summarized in Table S2. Titration was accomplished in 58, 78, 83 and 116 successive injections of BSA solution (5 μL each) with a constant stirring rate of 307 rpm and a time interval of 240–360 s between each injection. Measurements with number of injections exceeding 58 were accomplished by two series of injections, and the resulting ITC data were concatenate for analysis as a single file. The heat of dilution was measured, at the conditions of the respective experiment, by titration of BSA solution into the buffer and further subtracted from the heat of adsorption. 

**Data Analysis: SSIS Model.** Data were fit with the single set of independent binding sites (SSIS) model [30]. The SSIS model is based on the Langmuir adsorption theorem and allows one to define the binding constant as:

(1) $$K_{b}=\frac{\theta }{\left(1-\theta \right)\left[BSA\right]}$$

where θ is the fraction of the sites occupied by the protein and [*BSA*] is the concentration of free protein, which is connected with the total concentration of protein [*BSA*]_tot_ by equation:

(2) $${\left[BSA\right]}_{\mathrm{tot}}=\left[BSA\right]+N\theta \left[POL\right]$$

where N is the number of binding sites and [*POL*] is the total polymer concentration. The Langmuir equation assumes an equilibrium between the unoccupied binding sites of the macromolecule, the number of protein molecules in solution, and the occupied binding sites. 

**Figure 2:**
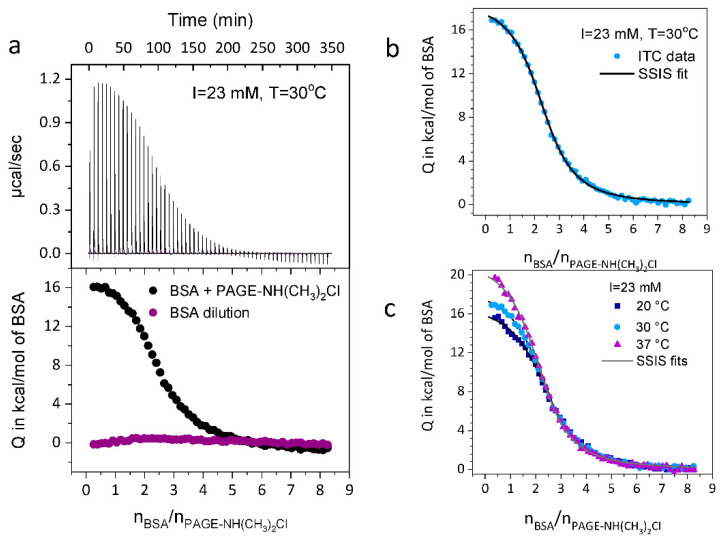
ITC-analysis exemplified for polymer **4**. (**a**) Top panel: the raw data from the titration of BSA to PAGE-NH(CH_3_)_2_Cl (black spikes) along with the raw dilution curve of BSA by buffer (purple spikes) at *T* = 30 °C and *I* = 23 mM. Lower panel: the integrated heats of each injection. (**b**) The respective SSIS-fits of curves obtained at *T* = 30 °C and (**c**) *T* = 20, 30 and 37 °C. The thermodynamic data derived from these fits are gathered in Table S6.

Fit parameters are the binding affinity (*K*_b_), the number of adsorption sites occupied by BSA (*N*_b_ *= ΘN*), and the calorimetric enthalpy (Δ*H*_ITC_). Figure 2 shows typical fits obtained for the linear cationic polyelectrolyte **4**. All data were corrected for the respective heat of dilution.

**Theory and Calculations.** In the following, a concise derivation and summary of the essential equations for further evaluation of the thermodynamic parameters gained from ITC measurements is given. 

***Master curve:*** We first turn to the dependence of the free energy of binding, Δ*G*_b_(*T*,*c*_s_), as the function of temperature. The purpose is to develop a master curve for the experimental data independent of any theoretical model. The binding constant, *K*_b_, can be measured precisely by isothermal titration calorimetry and is related to Δ*G*_b_(*T*,*c*_s_) by:

(3) $$\Delta G_{b}=-RTlnK_{b}$$

For most systems in which polyelectrolytes form complexes with proteins, a large specific heat, Δ*c_p_*, is found [5]. Thus, $\left|\Delta c_{p}\right|\gg \;\left|\Delta S_{b}\right|$ and both Δ*H*_b_ and Δ*S*_b_ exhibit a strong variation with temperature. Therefore, both Δ*H*_b_ and Δ*S*_b_ may become zero in the experimental range of temperatures. In this case, the entropy, Δ*S*_b_, is zero at temperature *T*_s_, whereas Δ*H*_b_ = 0 at temperature *T*_h_. As a consequence, the free energy, Δ*G*_b_, has an extremum and stays nearly constant around *T*_s_. In the vicinity of these characteristic temperatures, Δ*H*_b_ and Δ*S*_b_ can therefore be expanded to give [5,6,34,44]:

(4) $$\Delta H_{b}\left(T\right)≅\;\Delta H_{b}\left(T_{s}\right)+\Delta c_{p}\left(T-T_{s}\right)$$

and

(5) $$\Delta S_{b}\left(T\right)≅\Delta_{cp}ln\frac{T}{T_{s}}$$

The combination of both expressions leads to the well-known generalized van’t Hoff expression [45]:

(6) $$\Delta G_{b}\left(T\right)=\Delta H_{b,\mathrm{ref}}-T\Delta S_{b,\mathrm{ref}}+\Delta c_{p}\left[\left(T-T_{\mathrm{ref}}\right)-T\;\ln\left(\frac{T}{T_{\mathrm{ref}}}\right)\right]$$

With the reference temperature, *T*_ref_, given by *T*_s_, we obtain:

(7) $$\Delta G_{b}\left(T\right)=\Delta H_{b}\left(T_{s}\right)+\Delta c_{p}\left[\left(T-T_{s}\right)-T\;\ln\left(\frac{T}{T_{s}}\right)\right]$$

Equations (6) and (7) are exact if the conditions for Equations (4) and (5) are fulfilled. The specific heats, $\Delta c_{p}$, derived from fits of experimental data [31,34], however, are afflicted by a considerable error because this quantity depends critically on the curvature of the plots of $\Delta G_{b}$ as the function of temperature. 

Defining the reduced quantities:

(8) $$\Delta H_{\mathrm{red}}=\frac{\Delta H_{b}}{\Delta c_{p}T_{s}}$$

(9) $$\Delta S_{\mathrm{red}}=\frac{\Delta S_{b}}{\Delta c_{p}T_{s}}$$

we can rewrite Equation (7) as:

(10a) $$\frac{\Delta G_{b}}{\Delta c_{p}T_{s}}-\Delta H_{\mathrm{red}}\left(T_{s}\right)=\frac{T}{T_{s}}-1-\frac{T}{T_{s}}ln\frac{T}{T_{s}}$$

with

(10b) $$\Delta G_{\mathrm{red}}=\frac{\Delta G_{b}}{\Delta c_{p}T_{s}}$$

Hence, plots of $\Delta G_{\mathrm{red}}$ as the function of *T/T*_s_ should give a master curve for all systems where complex formation was measured in the vicinity of *T*_s_. Systems at the same reduced temperature, *T*_r_ = *T*/*T*_s_, are in corresponding states and can be compared directly.

With $\Delta T=T-T_{S}$ and series expansion of the logarithmic term in Equation (10a) around *T*_s_, we get:

$$\Delta H_{\mathrm{red}}\approx \Delta H_{\mathrm{red}}\left(T_{s}\right)+\frac{\Delta T}{T_{s}}$$

$$T\Delta S_{\mathrm{red}}\approx \frac{\Delta T}{T_{s}}+\frac{1}{2}{\left(\frac{\Delta T}{T_{s}}\right)}^{2}$$

(11) $$\Delta G_{\mathrm{red}}\approx \Delta H_{\mathrm{red}}\left(T_{s}\right)-\frac{1}{2}{\left(\frac{\Delta T}{T_{s}}\right)}^{2}$$

Equation (11) shows that $\Delta G_{\mathrm{red}}$ should exhibit a parabolic dependence on *T* for *T–T*_s_ < 20 K.

Therefore:

(12) $$\;\Delta H_{\mathrm{red}}-\Delta H_{\mathrm{red}}\left(T_{s}\right)\approx T\Delta S_{\mathrm{red}}-\frac{1}{2}{\left(\frac{\Delta T}{T_{s}}\right)}^{2}$$

Hence, for small Δ*T*/*T*_s_, the change of enthalpy is entirely compensated by the change of entropy. For temperatures around *T*_s_, plots of Δ*H*_b_ versus *T*Δ*S*_b_ will be linear with slope unity and an intercept, Δ*H*_b_(*T*_s_). For larger Δ*T*, the compensation of Δ*H*_b_ and Δ*S*_b_ is no longer complete. 

***Theory of complex formation***: The foregoing analysis of the data is purely phenomenological and can only serve as check of internal consistency of the data. Recently, we have developed a theoretical model which allows us the analysis of $\Delta G_{b}$ on salt concentration, *c_s_*, and temperature by a single expression [34]. Here, we only summarize the main equations necessary for the evaluation of data. Earlier work has clearly demonstrated that water release leads to an additional term in $\Delta G_{b}$, scaling linearly with *c*_s_ [1,39]. Hence [34]:

(13) $$\Delta G_{b}\left(T,c_{s}\right)=\;\mathrm{RT}\Delta n_{\mathrm{ci}}lnc_{s}-\mathrm{RT}0.036c_{s}\Delta w+\Delta G_{\mathrm{res}}$$

where Δ*w* describes the dependence of the free energy of binding on water release and $\Delta G_{\mathrm{res}}$ is a constant to be specified below [46]. Thus, *T* and *c*_s_ are the decisive variables that determine $\Delta G_{b}\left(T,c_{s}\right)$. Combination with Equations (4) and (5) above then leads to a closed expression for Δ*G*_b_(*T*,*c*_s_) [34]:

(14) $$\Delta G_{b}\left(T,c_{s}\right)=RT\Delta n_{\mathrm{ci}}lnc_{s}+\Delta H_{0}-\;T\Delta S_{0\;}+c_{s}\frac{d\Delta c_{p}}{dc_{s}}\left[T-T_{0}-T\ln\left(\frac{T}{T_{0}}\right)\right]$$

Here, the first term describes the contribution of counterion release to the free energy of binding. The quantity, Δ*n*_ci_, is the number of released counterions upon complex formation. The last term describes the effect of water release on Δ*G*_b_(*T*,*c*_s_) in terms of a new characteristic temperature, *T*_0_, whereas $\Delta H_{0}$ and $T\Delta S_{0\;}$ give the additional enthalpic and entropic contributions at *T = T*_0_. The respective enthalpy, $\Delta H_{b}$, and entropy, $\Delta S_{b}$, of binding read [34]:

(15) $$\Delta H_{b}\left(c_{s},T\right)=\Delta H_{0}+\frac{d\Delta c_{p}}{dc_{s}}c_{s}\left(T-T_{0}\right)\;$$

and

(16) $$\Delta S_{b}\left(c_{s},\;T\right)=\Delta S_{0}-\Delta n_{\mathrm{ci}}R\cdot \ln\left(c_{s}\right)+\frac{d\Delta c_{p}}{dc_{s}}c_{s}\ln\left(\frac{T}{T_{0}}\right)$$

The quantity, Δ*w*, follows as [34]

(17) $$\Delta w=\frac{\frac{d\Delta c_{p}}{dc_{s}}}{0.036R}\left(ln\frac{T}{T_{0}}+\frac{T_{0}}{T}-1\right)$$

showing that the effect of water release has an extremum at *T* = *T*_0_. For a positive coefficient, $\frac{d\Delta c_{p}}{dc_{s}}$, the effect of water release will hence increase the magnitude of Δ*G*_b_ and enhance the strength of binding. The parameter Δ*w* has been repeatedly determined from fits of Equation (13) to experimental data and interpreted as the number of released water molecules during complex formation [7,9,10]. Equation (17) shows, however, that this parameter depends sensitively on temperature and vanishes at *T* = *T*_0_. Hence, Δ*w* defies direct interpretation and must be viewed as a measure for the contribution of water release to the free energy of binding (see the discussion in [34] and further below).

Finally, the residual free energy, $\Delta G_{\mathrm{res}}$, is given by [34]:

(18) $$\Delta G_{\mathrm{res}}=\Delta H_{0}-T_{0}\Delta S_{0}$$

This contains all contributions to the free energy of binding such as, e.g., hydrogen bonding after removal of the parts related to counterion release and water release. It may also describe possible contributions to Δ*G*_b_ that are due to, e.g., conformational changes upon complex formation or salt bridges in the complex [46]. In total, Equation (14) provides a means to split up to the measured free energy of binding into three parts, namely: the effect of counterion release embodied in the logarithmic term; the effect of water release (cf. Equation (17)); and a term that covers all other effects. Thus, it allows us to quantify the role of water during complex formation. 

## 3. Results and Discussion

The polyelectrolytes **1**–**4** were synthesized by post-modification of PAGE (*M*_n_ = 12.1 kDa, *Ð* = 1.19) via thiol-ene reactions and purified by consecutive dialysis against salt solution and then pure water. ^1^H NMR and ^13^C NMR analysis indicated full conversion of the pendent allyl groups with the respective thiols. The opted post-modification strategy allowed the attainment of polyelectrolytes (Table S1) comprising the same number of around 100 repeating units (r.u.) for comparative binding studies with biomolecules. The lyophilized polyelectrolytes were dissolved in phosphate buffer (9 mM; pH 7.4) to achieve the requisite concentrations and used for the ITC experiments after ionic strength adjustment.

**Analysis by ITC**: The present analysis relies on precise data of the free energy of binding as the function of the two decisive variables temperature, *T*, and salt concentration, *c*_s._ In order to obtain the necessary accuracy, the subtraction of the heat of dilution must be done very carefully. We found that the anionic polysulfonate **1** binds weakly to BSA if the ionic strength is low (see Figures S12 and S13 in the Supplementary Materials), whereas the charge-neutral sulfonate-based polyzwitterion **2** does not allow evaluation via ITC under the applied conditions, suggesting the absence of any binding interaction with BSA (see Figure S14 in the Supplementary Materials). In contrast, a strong binding of BSA to the cationic polyamines **3** and **4** with pendant primary amine or tertiary dimethyl amine functionalities was observed. Figure 2 displays a typical example of the analysis of the binding of BSA to polymer **4**. All ITC-runs of polymers **3** and **4** are shown in Figures S15–S18, respectively, while Tables S4–S7 gather the resulting thermodynamic data. 

The fact that the anionic polysulfonate **1** binds only weakly to BSA at the ionic strength employed here comes as no surprise. In a previous investigation, we studied the binding of poly(acrylic acid) to HSA [19]. Here we found that binding occurred only at the lowest salt concentration of 20 mM, whereas a higher concentration of 50 mM already led to a very weak binding which was hardly measurable. The reason for this finding is located in the electrostatic repulsion of the polyelectrolyte and the like-charged BSA [12]. The thermodynamic analysis (Table S3) revealed an inconsistent number, *N* = 0.2–0.7, of bound polymer **1** chains per BSA within the applied range of temperature and salt concentration which, together with the low binding constant, *K*_b_, did not allow further detailed analysis of this data set. In the case of the apparently charge-neutral polymer **2**, we see the well-known stealth effect of zwitterionic polyelectrolytes, which do not interact strongly with proteins [47,48]. Due to the firm hydration of zwitterions, they hardly adsorb to biomolecules in solution or at biointerfaces [49]. Thus, flexible polyzwitterions, which exhibit limited electrostatic self-association capacity, combine an entropic with an enthalpic penalty derived from reduced chain flexibility and forced unfavourable dehydration upon complex formation with, e.g., proteins [50]. Hence, in the following, we shall discuss only the thermodynamic data obtained for the cationic polymers **3** and **4**.

The analysis by ITC leads to the binding constant, *K*_b_, the number of bound BSA-molecules per polymer chain, *N*, and the heat, Δ*H*_ITC_. The binding constant *K*_b_ can be converted into the free energy of binding via Equation (3) and provides the basis for the entire thermodynamic analysis. For polymer **3**, we find that *N* varies between 2.0 and 2.4, whereas *N* is 2.3–2.5 for polymer **4** (see Tables S4–S7). The slight variance of *N* with temperature and salt concentration may be due to the polydispersity of the polyelectrolyte chains. 

**Figure 3:**
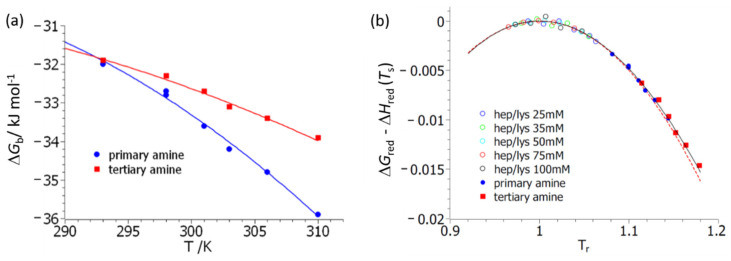
Analysis of the free energy, Δ*G*_b_, of complex formation between BSA and polymers **3** (primary amine) and **4** (tertiary amine), respectively, according to Equations (7) and (10a). (**a**) Fits of Equation (7) to experimental data. The parameters obtained from this fit are gathered in Table 1. (**b**) Analysis of the data in terms of the master curve Equation (10a). The reduced free energy of binding, Δ*G*_red_, is plotted against the reduced temperature, *T*_r_ *= T/T*_s_. The solid line denotes the exact results, whereas the dashed line displays the series expansion according to Equation (11). The hollow points refer to the data obtained for the system heparin/lysozyme studied in [33].

Figure 3 delineates the first step of the analysis of $\Delta G_{b\;}\left(T,c_{s}\right)\;$ with respect to temperature, *T*. Here, fits of Equation (7) to experimental data are shown. Excellent fits can be achieved for both polymers **3** and **4**. However, the resulting specific heat, Δ*c_p_*, is afflicted by an error of about 50%. The resulting parameters are gathered in Table 1. The maximal values of the free energy of binding, which equal Δ*H*_b_(*T*_s_), agree for both systems **3** and **4**, which may be traced back to their structural similarity on the molecular level. The other parameters, namely Δ*c_p_* and the temperature *T*_s_, at which the maximum value of Δ*G*_b_ is reached defy direct interpretation. However, the entire comparison suggests that both systems are directly comparable and interact with BSA in a similar manner. Moreover, this analysis provides the enthalpy and the entropy of reaction Δ*H*_b_ and Δ*S*_b_, which can later be compared to Equations (15) and (16), respectively.

Δ*H*_b_(*T*_s_): enthalpy of binding at *T = T*_s_ (Equation (7)); Δ*c_p_:* specific heat of binding determined from fits of Equation (7) to experimental data; *T*_s_: temperature where the entropy of binding vanishes; *T*_0_: characteristic temperature defined through Equation (14); Δ*n*_ci_: net number of released counterions (Equation (14)); dΔ*c_p_/*d*c*_s_: coefficient defining water release (Equation (14)); Δ*H*_0_, Δ*S*_0_: residual enthalpy and entropy of binding, respectively, as defined through Equation (14).

Figure 3b displays the same data but now plotted according to master curve Equation (10a). The solid line gives the exact expression of Equation (10a), whereas the dashed line displays the approximation of Equation (11). The hollow points show the respective thermodynamic data obtained for the binding of lysozyme to heparin studied and reported previously [33]. The calculation of the reduced free energy involves small differences of large numbers, and the plot in Figure 3b shows that the data have sufficient accuracy for a meaningful comparison. Figure 3b demonstrates that all data are described by the master curve Equation (10a). This in turn assures that Equations (4) and (5) present valid approximations, and further evaluation by Equation (14) is possible. 

The data gathered in Table 1 demonstrate that Δ*c_p_* is of appreciable magnitude, which in turn must lead to a strong compensation of entropy by enthalpy [5,6,34,44]. Equation (12) shows that this compensation must be total if Δ*T* is sufficiently small. This in turn is fulfilled if the reduced temperature, *T*_r_, is close to unity. 

**Figure 4:**
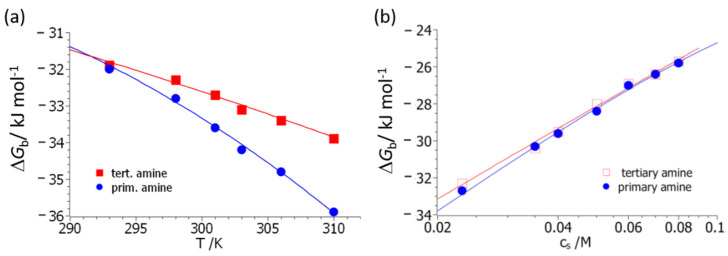
Comparison of theory and experiment. (**a**) The free energy of binding, Δ*G*_b_, is plotted against the temperature for polymers **3** (primary amine) and **4** (tertiary dimethyl amine). The salt concentration was fixed at *c*_s_ = 23 mM for all data. (**b**) The free energy of binding, Δ*G*_b_, is plotted against the salt concentration, *c*_s_, at a fixed temperature of 298 K for polymers **3** (primary amine) and **4** (tertiary dimethyl amine). The solid lines mark the fit by Equation (14) in both figures.

We now turn to the evaluation of the data according to Equation (14). Two variables determine the free energy of binding, Δ*G*_b_: the temperature, *T*, and the salt concentration, *c*_s_. The experimental Δ*G*_b_ (*T*,*c*_s_) is fitted by Equation (14) by the MathLab routine *cftool* to yield the parameters gathered in Table 1. As already discussed in [34], the parameters Δ*H*_0_ and Δ*S*_0_ can be determined securely because an entire set of data depending of *T* and *c*_s_ is fitted. The parameter dΔ*c_p_*/d*c*_s_ depends very much on the curvature of Δ*G*_b_ as the function of temperature and is therefore afflicted by a larger error; Δ*n*_ci_, on the other hand, can be determined very precisely [34]. Figure 4 displays the resulting fits, both as the function of *T* (Figure 4a) and of salt concentration, *c*_s_ (Figure 4b). Here it should be kept in mind that this rendition serves only for better visibility; all data are described by a single set of parameters. The interpretation of the experimental data in terms of Equation (14) differs from the fit according to Equation (10a) in a central point; Equation (14) contains the explicit dependence of Δ*G*_b_ on counterion release. Its removal therefore allows us to discuss the other factors leading to complex formation in a quantitative way.

Figure 4 demonstrates that a quantitative fit of all data can be achieved. The parameter Δ*n*_ci_ is slightly higher for polymer **3** compared to polymer **4**. It should be kept in mind that this quantity presents the net number of released counter- and co-ions. In all previous investigations, the parameter Δ*n*_ci_ was deduced from the slope of plots, as shown in Figure 4b. For both polymers **3** and **4**, these plots are linear within the limits of error. However, close inspection of Equation (14) reveals that this can only be the case if the data have been measured at temperatures close to *T*_0_ [34]. Figure 4b therefore shows small deviations from linearity, and the value of the parameter Δ*n*_ci_ obtained directly from the slope of the plot may be slightly different as compared to the one derived from Equation (14). The exact structure of the complex between BSA and polymers **3** and **4** cannot be deduced from the present set of data. In our previous analysis of the binding of HSA to poly(acrylic acid) [19], we found Δ*n*_ci_ ≅ 3, which is slightly higher than the values (2.8 for polymer **3** and 2.3 for polymer **4**) derived here (Table 1). 

As mentioned above, the fit according to Equation (14) allows us to split off the effect of counterion release on the free energy of binding, Δ*G*_b._ Thus, all other parameters refer to the remaining factors leading to complex formation. We first discuss the parameters that refer to the relation of Δ*G*_b_ (*T*,*c*_s_) to the release of water molecules during complex formation, namely, the last term in Equation (14). It is interesting to note that both systems have the same characteristic temperature, *T*_0_. This finding may be traced back to a similar binding strength between H_2_O and the polymer chains. The main difference resides in the parameter dΔ*c_p_/*d*c*_s_, which measures the gain of free energy with increasing salt concentration for a given temperature difference, *T–T*_0_. For both polymers, this coefficient is positive, and water release will therefore increase the free energy of binding. In the case of polymer **3**, this increase is larger by a factor of nearly 6 as compared to polymer **4**. Hence, this factor reflects the fact that the magnitude of Δ*G*_b_ is increasing much more for polymer **3** as compared to polymer **4** when increasing the temperature from *T*_0_ to 310 K (see Figure 4a). In other words, the effect of water release on Δ*G*_b_ with raising temperature is increasing much more for polymer **3** than for polymer **4**. 

As outlined previously [34], the quantity, Δ*w* (see Equation (17)), can be interpreted in terms of the solute partitioning model (SPM) by Record et al. [39,40]. The SPM describes the interaction of the ions with the protein in aqueous solution by a combination of an ion-specific interaction and the non-specific lowering of the water activity by the salt ions. The former contribution is directly related to the Hofmeister series [40]. During complex formation, Δ*B*_H2O_ water molecules will be released. The relation of the SPM to the present model is given by [34]:

(19) $$\Delta w\;=\;\frac{1}{2}\left(K_{p,+\;}+\;K_{p,-\;}-2\right)\Delta B_{H2O}$$

where partition coefficient $K_{p,+}=(m_{+}^{\mathrm{loc}}$/$m_{+}^{\mathrm{bulk}}$) describes the distribution of the cations between the hydrate and the bulk water. Thus, $m_{+}^{\mathrm{loc}}$ is the molality of the cations in the hydrated shell, whereas $m_{+}^{\mathrm{bulk}}$ is the respective quantity in bulk. The anions are distributed in the same way characterized by the partition coefficient, $K_{p,-}$. Analogously, the partition coefficient, $K_{p,-}$, refers to the partition of the anions between the hydrate shell and the bulk water. For salt ions in the middle of the Hofmeister series, these partition coefficients are approximately unity, and the contribution of water release to Δ*G*_b_ is small. At *T = T*_0_, Δ*w* vanishes and the present model predicts a parabolic dependence on *T–T*_0_ according to Equation (17). The coefficient dΔ*c_p_/*d*c*_s_ is a quantitative measure for the effect of water release at a certain *T* − *T*_0_. 

The data gathered in Table 1 refer to two different polyelectrolytes interacting with the same protein, BSA. Hence, the differences seen for polymer **4** compared to polymer **3** must be traced back to a differential local interaction of both polymers with BSA. Because the effects seen here cannot be solely attributed to the hydration of the protein, it necessarily implies that it is the hydration of the polymers which causes the observed difference. This is reasonable as dimethyl amino groups are generally less hydrated compared to primary amino groups due to the replacement of two hydrogen bond donating substituents on the nitrogen with methyl groups. Thus, the resulting parameters gathered in Table 1 can be explained as follows: First of all, the number of released counterions, Δ*n*_ci_, is slightly but significantly higher for polymer **3**. This may be due to the shorter distance between the cationic charge and the surface of the protein in the case of polymer **3,** whereas there may be a steric hindrance due to the methyl groups in the case of polymer **4**. Concomitantly, a closer interaction between the primary amine group with the surface of BSA is followed by a stronger release of water, as measured by the coefficient dΔ*c_p_/*d*c*_s_. As mentioned above, the quantities Δ*H*_0_ and Δ*S*_0_ refer to the residual free energy after splitting off both the contributions of counterion and water release. The data gathered in Table 1 show that Δ*H*_0_ is small for both polymers **3** and **4**, and the residual free energy is mainly entropic. 

The discussion of the parameters deriving from the fits to Equation (14) allow us to discern between the main contributions to the free energy of binding, namely counterion release and hydration and their relation to ionic strength; counterion release is dominant at low ionic strength and diminishes logarithmically with increasing salt concentration. The contribution originating from hydration, however, scales linearly with salt concentration and therefore increases strongly with increasing salinity of the solution. At physiological ionic strength, hydration may therefore become the leading term in Δ*G*_b_ if the temperature differs significantly from *T*_0_. The parameters gathered in Table 1 therefore allow us to extrapolate Δ*G*_b_ to conditions where ITC-measurements have become insecure.

Finally, we discuss the marked enthalpy–entropy compensation as embodied in Equation (14) [34]. First, it should be noted that the heat, Δ*H*_ITC_, measured directly in the ITC-experiment is not necessarily the heat of reaction, Δ*H*_b_. This fact has already been observed in early work [45] and can be explained by linked equilibria (see, e.g., the discussion of this point in [34]): If complex formation is connected to the uptake or release of protons, the equilibration of the pH by the buffer will lead to an additional heat effect caused by the uptake/release of protons by the buffer. This heat is not related to complex formation but is also contained in Δ*H*_ITC_. Experiments with buffers differing in the heat of protonation have shown this clearly, and the true heat of reaction can only be obtained by extrapolation of Δ*H*_ITC_ to a vanishing heat of protonation of the buffer [34]. We therefore use only the binding enthalpies, Δ*H*_b_, derived from fits of Equation (7) to the experimental data.

**Figure 5:**
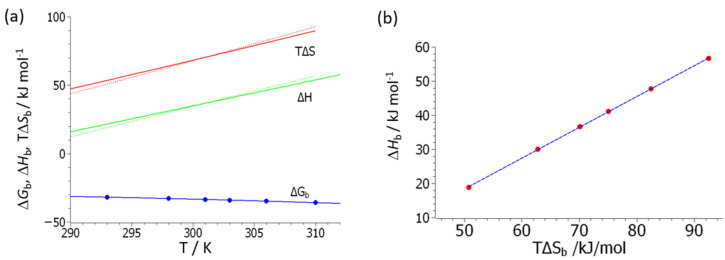
Enthalpy–entropy compensation for the data obtained from polymer **3** (primary amine). (**a**) The blue circles denote the measured free energy of binding, whereas the blue solid line gives the respective fit by Equation (14). The solid green and red lines give the enthalpy Equation (15) and the entropy multiplied by *T* as obtained from Equation (16), respectively. The respective dashed lines denote the enthalpy Equation (4) and the entropy Equation (5) deriving from the fit of Equation (7) to the experimental data. (**b**) The enthalpy, Δ*H*_b_ (cf. Equation (4)), obtained from the fit of the experimental data according to Equation (7) is plotted against *T*Δ*S*_b_ (Equation (5)). The slope of the dashed line is 0.9, indicating an incomplete compensation of enthalpy by entropy. See text for further explanation.

The strong enthalpy–entropy compensation can now be discussed in more detail using Equations (15) and (16). Figure 5 displays a comparison between the enthalpy and entropy obtained from the fits of the generalized van’t Hoff equation (7) to the experimental data obtained for polymer **3** (primary amine); the respective comparison for polymer **4** is shown in Figure S19 of the Supplementary Materials. The dashed lines in Figure 5a display these data, whereas the full lines show the results of Equations (15) and (16) using the parameters gathered in Table 1. Full agreement within the prescribed limits of error is seen.

The compensation of enthalpy by entropy is not complete, as can be seen from Figure 5b and Figure S19b. Here we plot the enthalpy, Δ*H*_b_, calculated by Equation (4) against *T*Δ*S*_b_ (Equation (5)), where the respective constants Δ*H*_b_(*T*_s_) and Δ*c_p_* have been determined by a fit of Equation (7) to the experimental data obtained on polymer **3**. The slope of the dashed line in Figure 5b is 0.9, which indicates that the enthalpy of binding is not entirely compensated by entropy. This finding is due to the fact that the data for polymer **3** have been taken at a rather high reduced temperature of *T*_r_ > 1.1. Therefore, the term scaling with Δ*T*^2^ in Equation (12) is no longer negligible and the enthalpy–entropy compensation no longer complete, as has been found for the system heparin/lysozyme (see the open circles in Figure 3b and the discussion in [33]). It should be noted, however, that the generalized van’t Hoff fit Equation (7) is afflicted by a considerable error and a fit according to Equation (14) is by far better and more stable.

## 4. Conclusions

We have presented a thermodynamic analysis of the interaction of linear polyelectrolytes with bovine serum albumin (BSA). All polyelectrolytes are derived from the same main chain by polymer-analogous reaction (see Figure 1) and only differ regarding the chemical group presenting the charge and the sign of the charge. We found the anionic polymer **1** to only weakly interact while the zwitterionic polymer **2** does not interact at all with BSA under the applied conditions. A strong binding, however, was found for the cationic polymers **3** and **4**, which could be analysed in detail by ITC. The analysis was performed using Equation (14), which describes the free energy of binding Δ*G*_b_ as the function of temperature, *T*, and salt concentration, *c*_s_. The underlying model splits Δ*G*_b_ into a term related to counterion release and a term related to water release [34]. The main result of this analysis is the observation that the release of water, as expressed in the coefficient dΔ*c_p_/*d*c*_s_, is more important for polymer **3**, which bears primary amino groups (cf. Table 1). We explain this finding by a closer contact of this polymer with BSA in the complex when compared to polymer **4** comprising bulkier tertiary amino groups. In addition, the latent hydrophobicity of the tertiary dimethyl amine groups in polymer **4** compared to the more hydrophilic primary amines generally results in a weaker hydration which may contribute to the overserved lower degree of released water upon complex formation between BSA and polymer **4**. The entire discussion of the data in terms of Equation (14) demonstrates that the binding strength, Δ*G*_b_, can be dissected quantitatively into different contributions that depend on temperature and salt concentration. In this way, a full understanding of complex formation between polyelectrolytes and proteins can be achieved. 

Future studies will focus on the investigation of synthetic polycations with additional biocompatibilising pendant groups such as charge-neutral zwitterions or oligoethylene glycols.

## Figures and Tables

**Table 1:** Thermodynamic parameters obtained from fits of Equations (7) and (14) with an estimate of their error margins.

Polymer	Δ*H*_b_(*T*_s_) [kJ mol^−1^]	Δ*c_p_* [kJ mol^−1^K^−1^]	*T*_s_ [K]	*T*_0_ [K]	Δ*n*_ci_	$\frac{dc_{p}}{dc_{s}}$ [kJ mol^−1^K^−1^M^−1^]	Δ*H*_0_ [kJ mol^−1^]	Δ*S*_0_ [kJ K^−1^mol^−1^]
**3**	−30 ± 5	2.2 ± 0.5	271 ± 4	285 ± 3	2.76 ± 0.03	82 ± 8	6.4 ± 0.5	0.044 ± 0.004
**4**	−30 ± 5	0.9 ± 0.5	263 ± 4	285 ± 3	2.27 ± 0.03	14 ± 2	−1.7 ± 0.2	0.031 ± 0.003

Δ*H*_b_(*T*_s_): enthalpy of binding at *T* = *T*_s_ (Equation (7)); Δ*c_p_*: specific heat of binding determined from fits of Equation (7) to experimental data; *T*_s_: temperature where the entropy of binding vanishes; *T*_0_: characteristic temperature defined through Equation (14); Δ*n*_ci_: net number of released counterions (Equation (14)); dΔ*c_p_*/d*c*_s_: coefficient defining water release (Equation (14)); Δ*H*_0_, Δ*S*_0_: residual enthalpy and entropy of binding, respectively, as defined through Equation (14).

## Supplementary Materials

The following are available online at https://www.mdpi.com/article/10.3390/biom11091377/s1. Polyelectrolyte characterization (GPC and NMR data) as well as ITC measurement conditions with raw data and basic analysis can be found in the Supplementary Information. Figure S1: GPC profile of the precursor polymer PAGE in THF as the eluent applying PS-standards, Figure S2: ^1^H-NMR of PAGE (CDCl_3_, 400 MHz), Figure S3: ^13^C-NMR of PAGE (CDCl_3_, 175 MHz), Figure S4: ^1^H-NMR (400 MHz, D_2_O) of polymer **1** (PAGE-SO_3_Na), Figure S5: ^13^C-NMR (176 MHz, D_2_O) of polymer **1** (PAGE-SO_3_Na), Figure S6: ^1^H-NMR (400 MHz, D_2_O) of polymer **2** (PAGE-N^+^(CH_3_)_2_-(CH_2_)_3_-SO_3_^−^), Figure S7: ^13^C-NMR (176 MHz, D_2_O) of polymer **2** (PAGE-N^+^(CH_3_)_2_-(CH_2_)_3_-SO_3_^−^), Figure S8: ^1^H-NMR (400 MHz, D_2_O) of polymer **3** (PAGE-NH_3_Cl), Figure S9: ^13^C-NMR (176 MHz, D_2_O) of polymer **3** (PAGE-NH_3_Cl), Figure S10: ^1^H-NMR (400 MHz, D_2_O) of polymer **4** (PAGE-NHMe_2_Cl), Figure S11: ^13^C-NMR (176 MHz, D_2_O) of polymer **4** (PAGE-NHMe_2_Cl), Table S1: Overview of molecular weight of functional polymers **1**–**4**, Table S2: Summary of used concentrations and measurement conditions in ITC experiments, Figure S12: ITC data of the adsorption of BSA onto polymer **1** (PAGE-SO_3_Na) (black curves) with the corresponding heats of dilution of BSA (blue curves) at pH = 7.4, *I* = 23 mM and (a) *T* = 25 °C, (b) *T* = 30 °C, (c) *T* = 37 °C. (d) Binding isotherms corrected for the heat of dilution at *T* = 25, 30, 37 °C and 23 mM, Figure S13: ITC data of adsorption of BSA onto polymer **1** (PAGE-SO_3_Na) (black curves) with the corresponding heats of dilution of BSA (blue curves) at pH = 7.4, *T* = 25 °C and (a) *I* = 23 mM, (b) *I* = 35 mM, (c) *I* = 40 mM, (d) *I* = 50 mM, (e) *I* = 80 mM. (d) Binding isotherms corrected for the heat of dilution at *T* = 25 °C for all ionic strengths, Table S3: Thermodynamic parameters of the binding between BSA (c = 0.4 mM) and polymer **1** (PAGE-SO_3_Na) (c = 0.03 mM) obtained from fitted binding isotherms, Figure S14: ITC data of adsorption of BSA (c = 0.37 mM) onto polymer **2** (PAGE-N^+^(CH_3_)_2_-(CH_2_)_3_-SO_3_^−^) (black curves) (c = 0.01 mM) with the corresponding heats of dilution (green curves) of BSA at pH = 7.4, *I* = 23 mM, Figure S15: ITC data of adsorption (black curves) of BSA (c = 0.37 mM) onto polymer **3** (PAGE-NH_3_Cl) (c = 0.01 mM) with the corresponding heats of dilution (red curves) of BSA at pH = 7.4, *I* = 23 mM and (a) *T* = 20 °C, (b) *T* = 25 °C, (c) *T* = 28 °C, (d) *T* = 30 °C, (e) *T* = 33 °C (f) *T* = 37 °C. (g) Binding isotherms corrected for the heat of dilution at *T* = 20, 25, 28, 30, 33, 37 °C and *I* = 23 mM, Table S4: Thermodynamic parameters of binding between BSA (c = 0.37 mM) and polymer **3** (PAGE-NH_3_Cl) (c = 0.01 mM) obtained from fitted isotherms based on a temperature series; Figure S16: ITC data of the adsorption (black curves) of BSA (c = 0.4 mM) onto polymer **3** (PAGE-NH_3_Cl) (c = 0.007 mM) with the corresponding heats of dilution (red curves) of BSA at pH = 7.4, *T* = 25 °C and (a) *I* = 23 mM, (b) *I* = 35 mM, (c) *I* = 40 mM, (d) *I* = 50 mM, (e) *I* = 60 mM, (f) *I* = 70 mM, (g) *I* = 80 mM. (h) Binding isotherms corrected for the heat of dilution at *T* = 25 °C and *I* = 23, 35, 40, 50, 60, 70, 80 mM, Table S5: Thermodynamic parameters of binding between BSA (c = 0.4 mM) and polymer **3** (PAGE-NH_3_Cl) (c = 0.007 mM) obtained from fitted isotherms based on the ionic strength series, Figure S17: ITC data of adsorption (black curves) of BSA (c = 0.37 mM) onto polymer **4** (PAGE-NH(CH_3_)_2_Cl) (c = 0.01 mM) with the corresponding heats of dilution (violet curves) of BSA at pH = 7.4, *I* = 23 mM and (a) *T* = 20 °C, (b) *T* = 25 °C, (c) *T* = 28 °C, (d) *T* = 30 °C, (e) *T* = 33 °C, (f) *T* = 37 °C. (g) Binding isotherms corrected for the heat of dilution at *T* = 20, 25, 28, 30, 33, 37 °C and *I* = 23 mM, Table S6: Thermodynamic parameters of binding between BSA (c = 0.37 mM) and polymer **4** (PAGE-NH(CH_3_)_2_Cl) (c = 0.01 mM) obtained from fitted isotherms based on a temperature series, Figure S18: ITC data of the adsorption (black curves) of BSA (c = 0.4 mM) onto polymer **4** (PAGE-NH(CH_3_)_2_Cl) (c = 0.01 mM) with the corresponding heats of dilution (violet curves) of BSA at pH = 7.4, *T* = 25 °C and (a) *I* = 23 mM, (b) *I* = 35 mM, (c) *I* = 40 mM, (d) *I* = 50 mM, (e) *I* = 60 mM, (f) *I* = 70 mM, (g) *I* = 80 mM. (h) Binding isotherms corrected for the heat of dilution at *T* = 25 °C and *I* = 23, 35, 40, 50, 60, 70, 80 mM. Table S7: Thermodynamic parameters of binding between BSA (c = 0.4 mM) and polymer **4** (PAGE-NH(CH_3_)_2_Cl) (c = 0.01 mM) obtained from fitted isotherms based on the ionic strength series. Figure S19: Enthalpy–entropy compensation for the data obtained from polymer **4** (tertiary amine). (a): The blue circles denote the measured free energy of binding, whereas the blue solid line gives the respective fit by Equation (14). The solid green and red lines give the enthalpy Equation (15) and the entropy multiplied by *T*, as obtained from Equation (16), respectively. The respective dashed lines denote the enthalpy Equation (4) and the entropy Equation (5) deriving from the fit of Equation (7) to the experimental data. (b) The enthalpy Δ*H*_b_ (cf. Equation (4)) obtained from the fit of the experimental data according to Equation (7) is plotted against *T*Δ*S*_b_ (Equation (5)). The slope of the dashed line is 0.88, indicating an incomplete compensation of enthalpy by entropy. See text for further explanation.
