# Supplementary material for: Interaction of Linear Polyelectrolytes with Proteins: Role of Specific Charge–Charge Interaction and Ionic Strength
Source: Biomolecules. 2021 Sep 17;11(9):1377. doi: 10.3390/biom11091377 (PMC8472085; doi:10.3390/biom11091377)
Supplement: Supplementary file 1 [file biomolecules-11-01377-s001.zip › biomolecules-1363948-supplementary.pdf]

Interaction of linear polyelectrolytes with proteins: Role of specific charge-charge interaction and ionic strength

Julia Bukala[a], Prabhusrinivas Yavvari[a], Jacek J. Walkowiak[a,1] Matthias Ballauff[a,] Marie Weinhart [a,b*]*

[a] Department of Chemistry and Biochemistry, Freie Universität Berlin, Takustrasse 3, 14109 Berlin, Germany

[b] Institute of Physical Chemistry and Electrochemistry, Leibniz Universität Hannover, Callinstr. 3A, 30167 Hannover

Supplementary Information

Content

1. Polyelectrolyte Characterization 2

1.1 GPC chromatogram of PAGE 2

1.2 NMR-Data of PAGE and polymers **1-4** 3

1.3 Summary of molecular characteristics of PAGE and polymers **1-4** 8

2. ITC measurement conditions 8

3. Thermodynamic parameters obtained from ITC measurements 9

3.1 Representative ITC curves of polymer **1** binding to BSA and extracted thermodynamic parameters 9

3.2 Representative ITC curves of polymer **2** with BSA 11

3.3 Representative ITC curves of polymer **3** binding to BSA and extracted thermodynamic parameters 12

3.4 Representative ITC curves of polymer **4** binding to BSA and extracted thermodynamic parameters 16

---

[1] Present address: Aachen-Maastricht Institute for Biobased Materials, Maastricht University Brightlands Chemelot Campus, Urmonderbaan 22, 6167 RD Geleen, The Netherlands

1. Polyelectrolyte Characterization

1.1 GPC chromatogram of PAGE

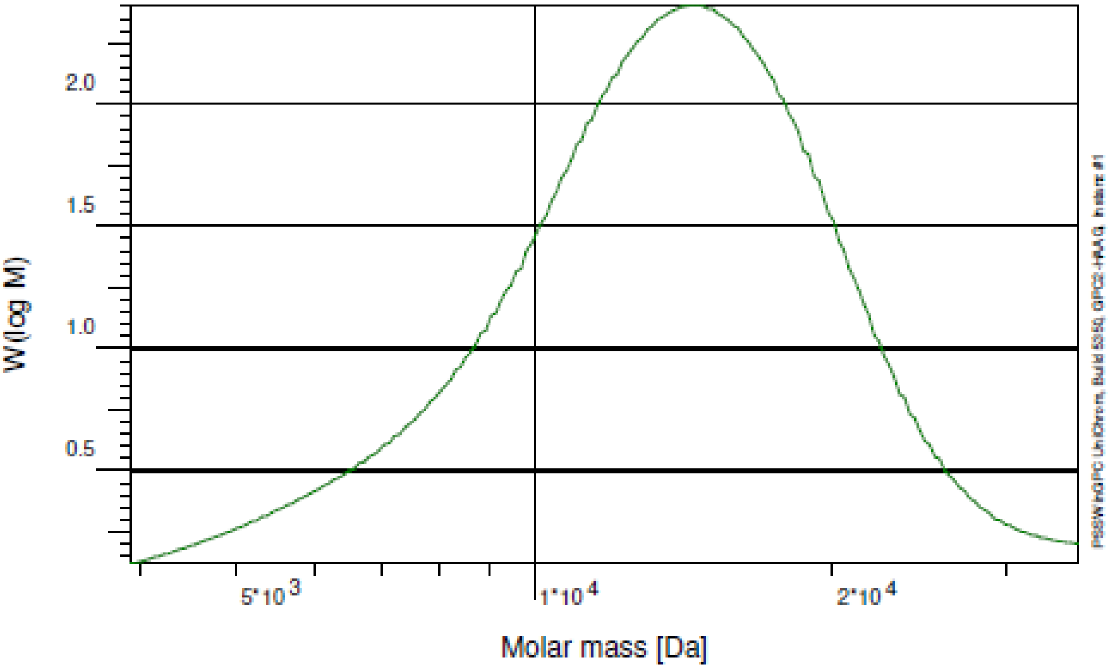

**Probe :**	Vial 5: PS85 - 1		
**Integration von :**	Mittwoch 23.01.19 15:13:36		21.745 ml
**Integration bis :**	Mittwoch 23.01.19 15:16:43		24.862 ml
**Kalibration :**	Polystyrol 180716 80bar.CAL	**Eluent :**	THF
**MHK - A (Kal.):**	0.000E+0	**MHK - K (Kal.):**	1.000E+0 ml/g
**Int.Stand.-K :**	28.730 ml	**Int.Stand.-M :**	28.670 ml
**Pumpe :**	PSS SECcurity	**Flussrate :**	1.000 ml/min
**Konzentration :**	3.000 g/l	**Injektvolumen :**	100.000 ul
**Säule 1 :**	PL Gel 5µm Vorsäule	**Temperatur :**	25.000 °C
**Säule 2 :**	PL Gel 5µm mix-C	**Temperatur :**	0.000 °C
**Säule 3 :**	PL Gel 5µm mix-C	**Temperatur :**	0.000 °C
**Säule 4 :**	PL Gel 5µm mix-C	**Temperatur :**	0.000 °C
**Detektor 1 :**	I1: RID 1, RI Signal	**Versatz :**	0.000 ml
**Detektor 2 :**	I1: IsoPump 1, Pressure	**Versatz :**	0.000 ml
**Operateur :**	Administrator	**Messintervall :**	1.000 sec

I1: RID 1, RI Signal

**Mn :**	1.2108e4	g/mol
**Mw :**	1.4361e4	g/mol
**Mz :**	1.6617e4	g/mol
**Mv :**	0.000000	g/mol
**D :**	1.1861e0	
**[n]:**	0.000000	ml/g
**Vp :**	2.3018e1	ml
**Mp :**	1.4450e4	g/mol
**FI :**	1.0358e4	ml*V
**< 3918**	0.00	
**w% :**	100.00	
**> 35328**	0.00	

Figure S1. GPC profile of the precursor polymer PAGE in THF as the eluent applying PS-standards.

1.2 NMR-Data of PAGE and polymers **1-4**

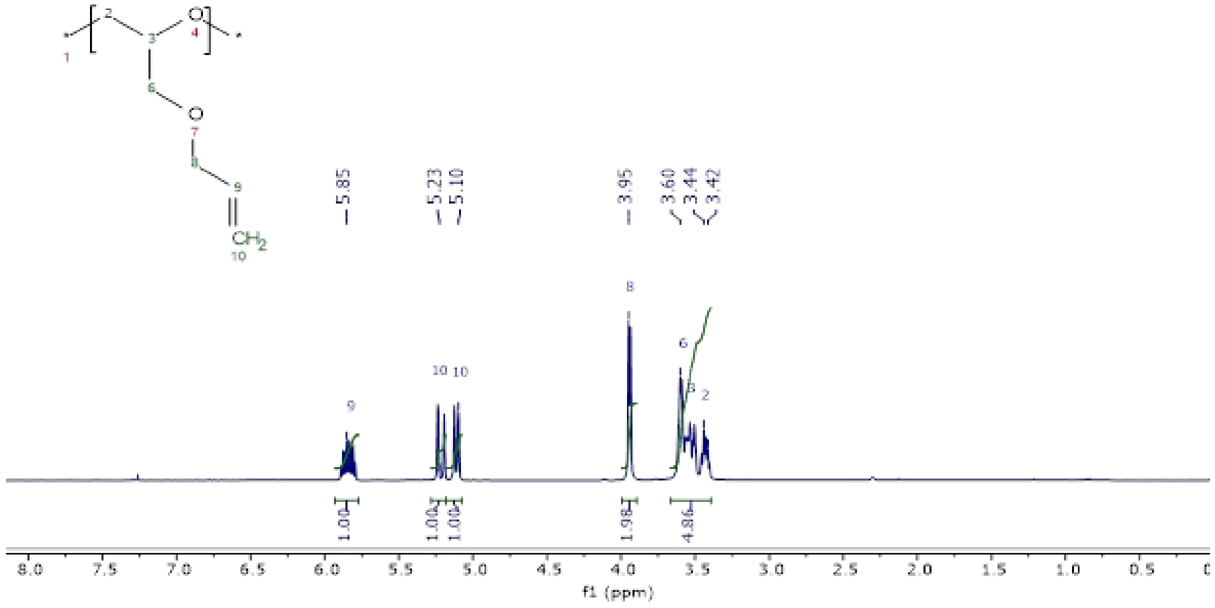

Figure S2. ^{1}H-NMR of PAGE ($CDCl_3$, 400 MHz).

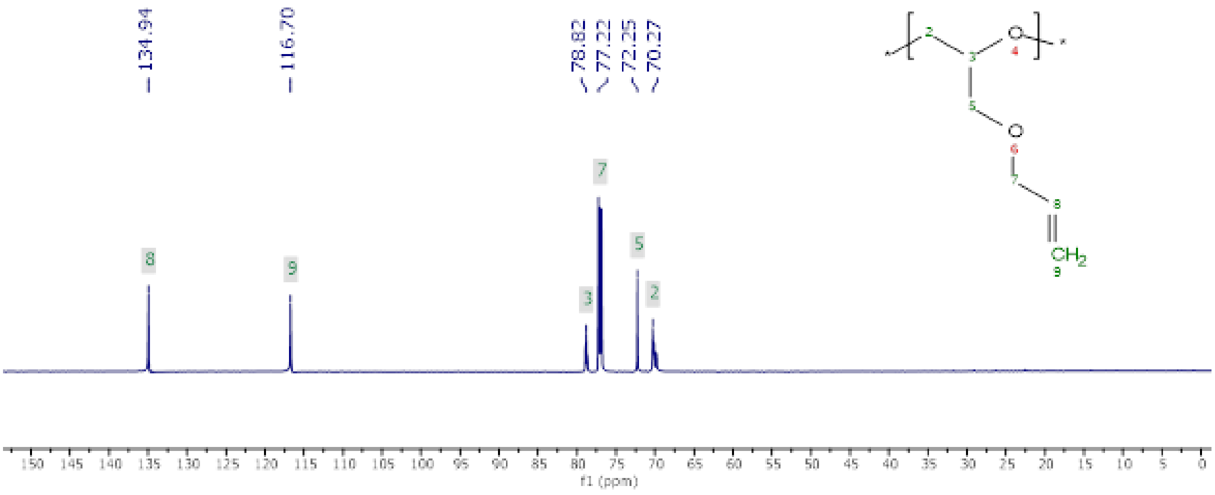

Figure S3. ^{13}C-NMR of PAGE ($CDCl_3$, 175 MHz).

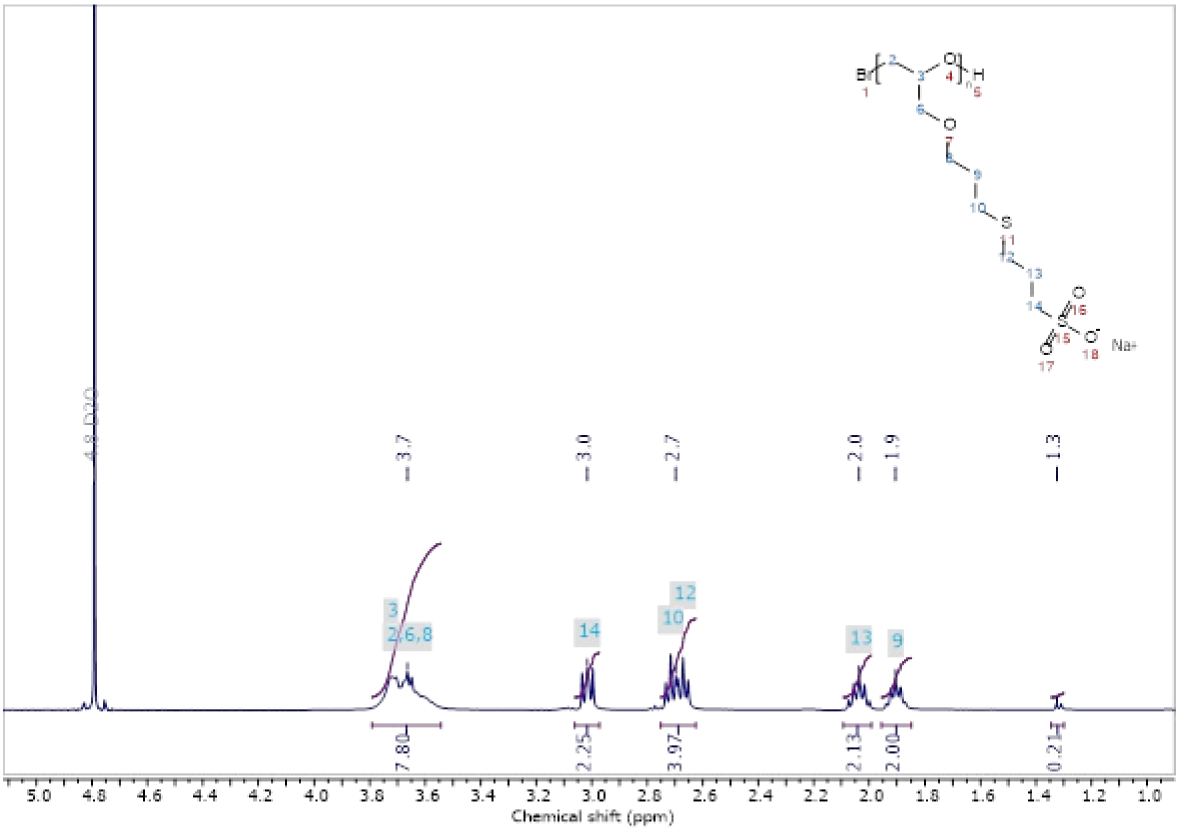

Figure S4. ^{1}H-NMR (400 MHz, D_2O) of polymer **1** (PAGE-SO_3Na).

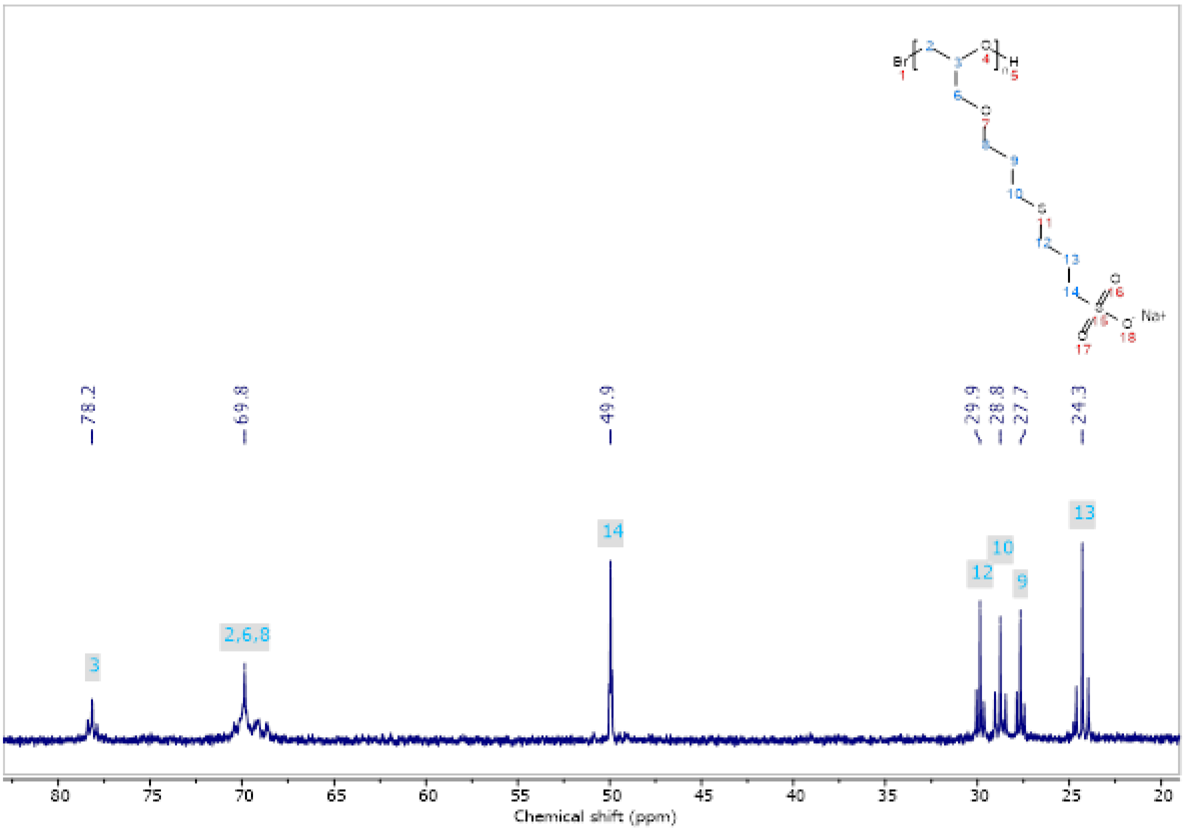

Figure S5. ^{13}C-NMR (176 MHz, D_2O) of polymer **1** (PAGE-SO_3Na).

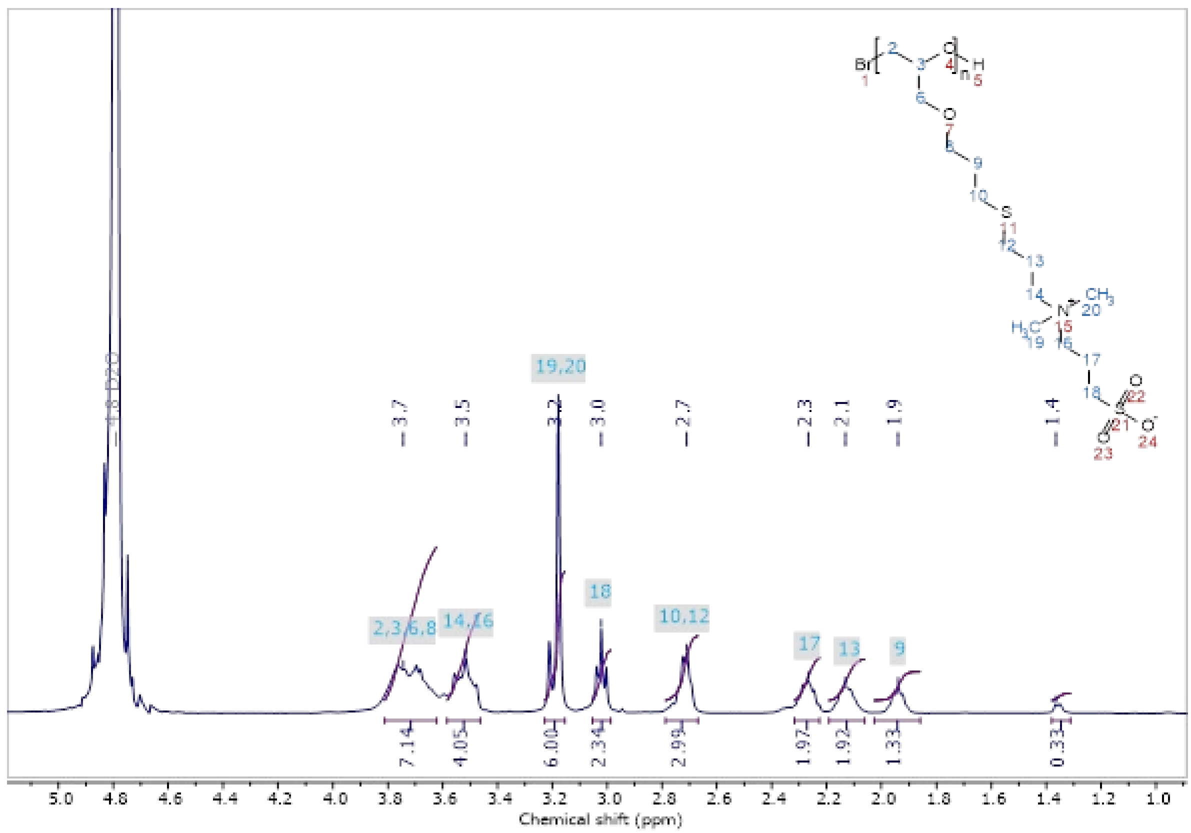

Figure S6. ^{1}H-NMR (400 MHz, D_2O) of polymer **2** (PAGE-$N^{+}(CH_3)_2$-$(CH_2)_3$-SO_3^{-}).

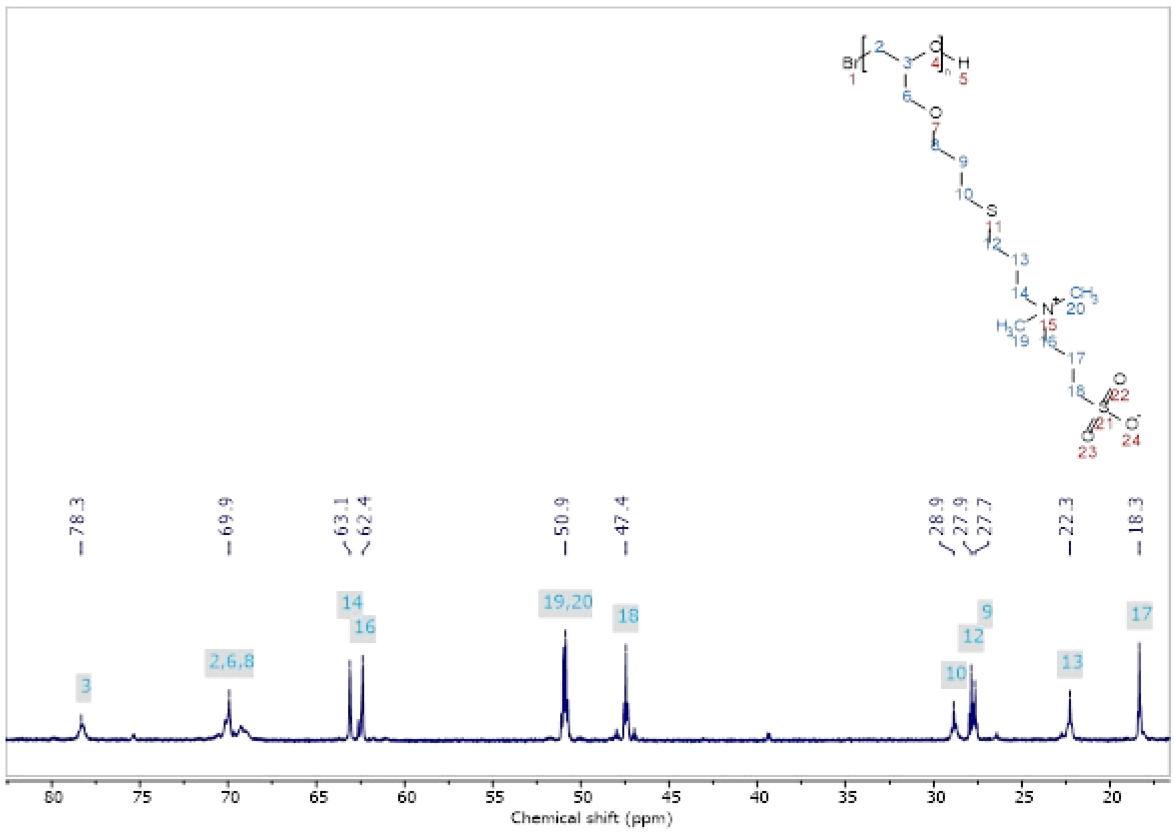

Figure S7. ^{13}C-NMR (176 MHz, D_2O) of polymer **2** (PAGE-$N^{+}(CH_3)_2$--$(CH_2)_3$-SO_3^{-}).

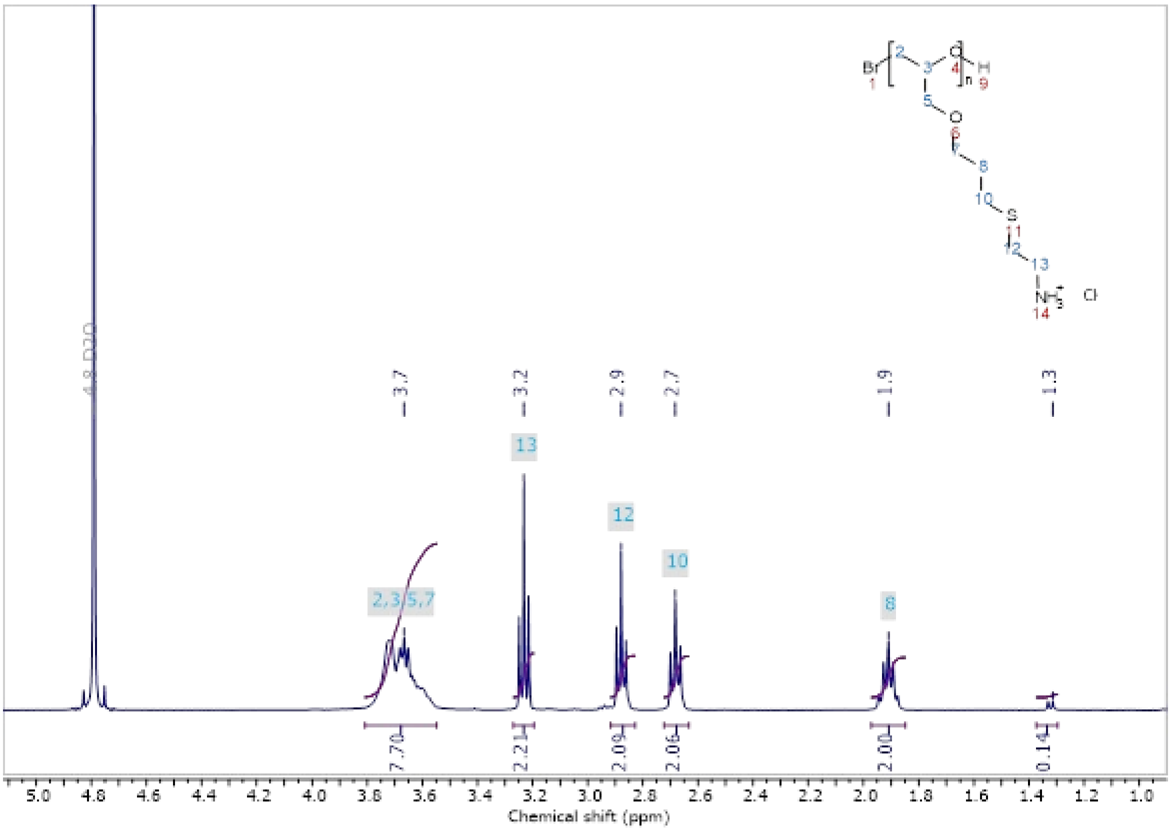

Figure S8. ^{1}H-NMR (400 MHz, D_2O) of polymer **3** ($PAGE-NH_3Cl$).

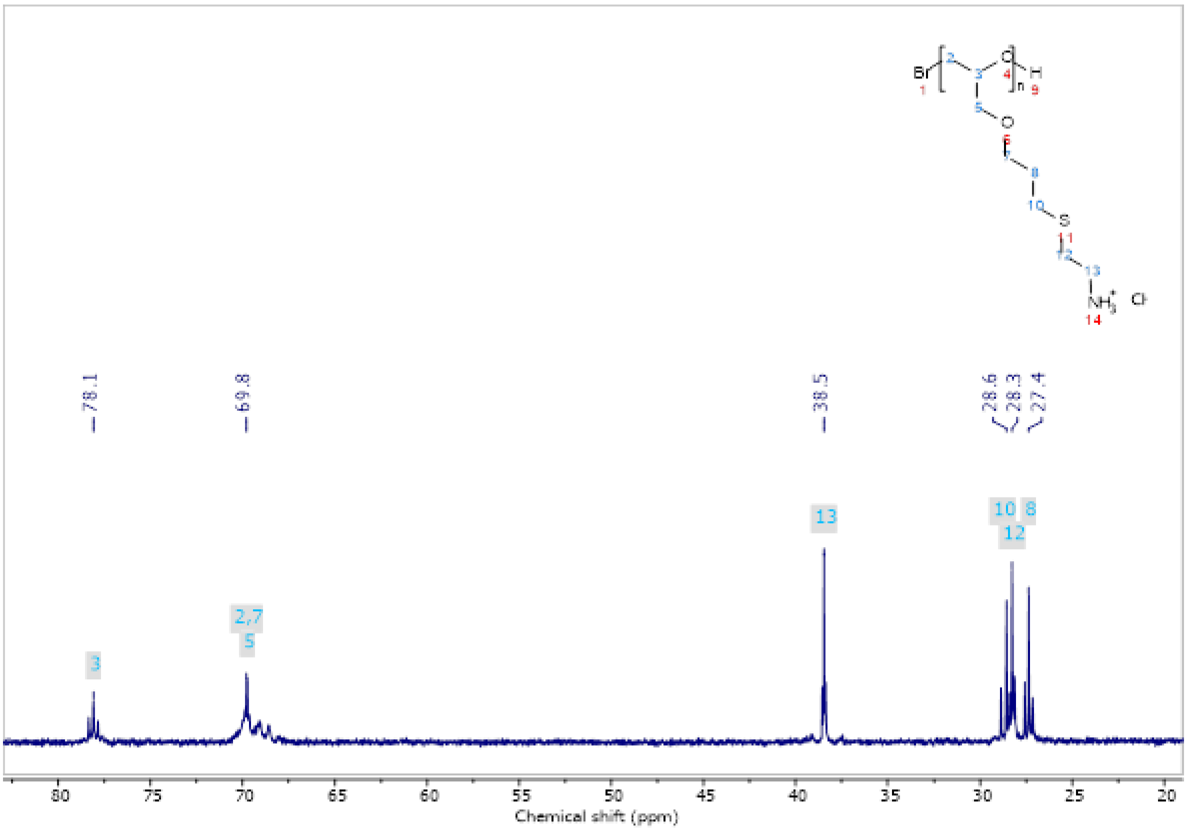

Figure S9. ^{13}C-NMR (176 MHz, D_2O) of polymer **3** ($PAGE-NH_3Cl$).

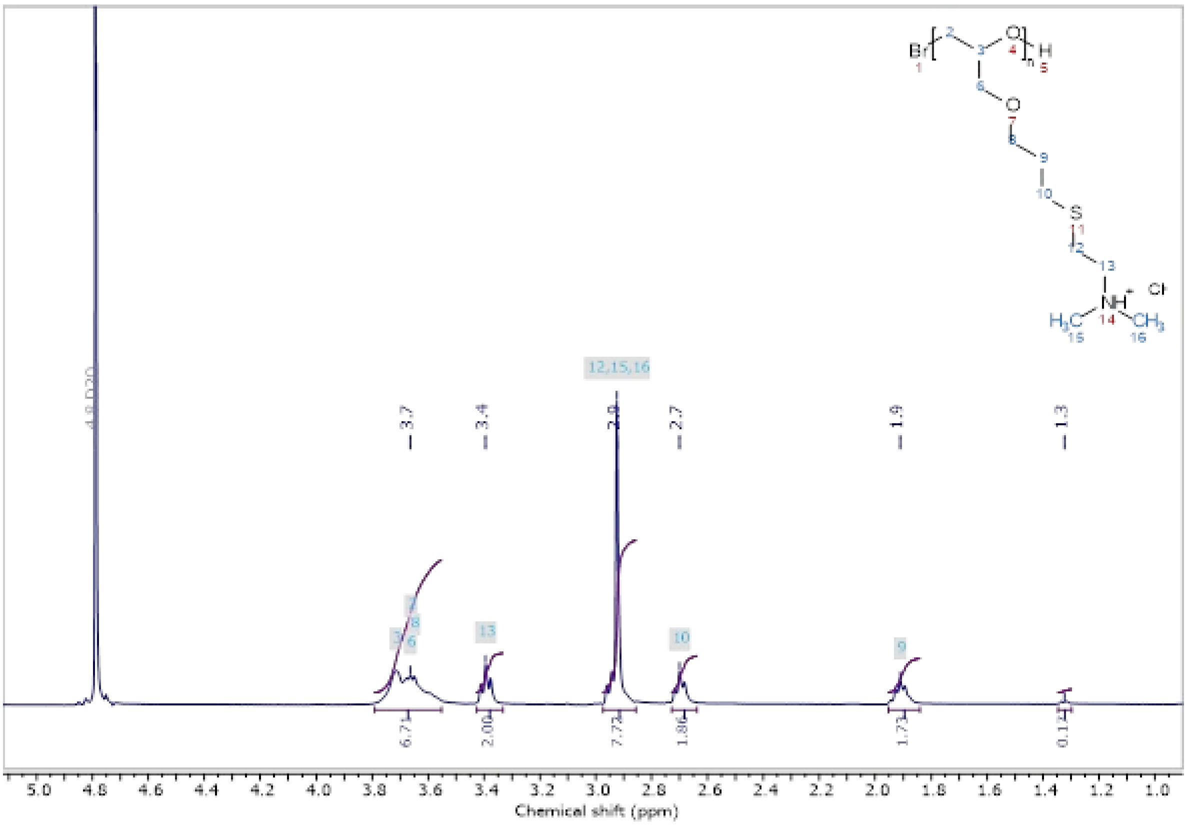

Figure S10. ^{1}H-NMR (400 MHz, D_2O) of polymer **4** (PAGE-$NHMe_2Cl$).

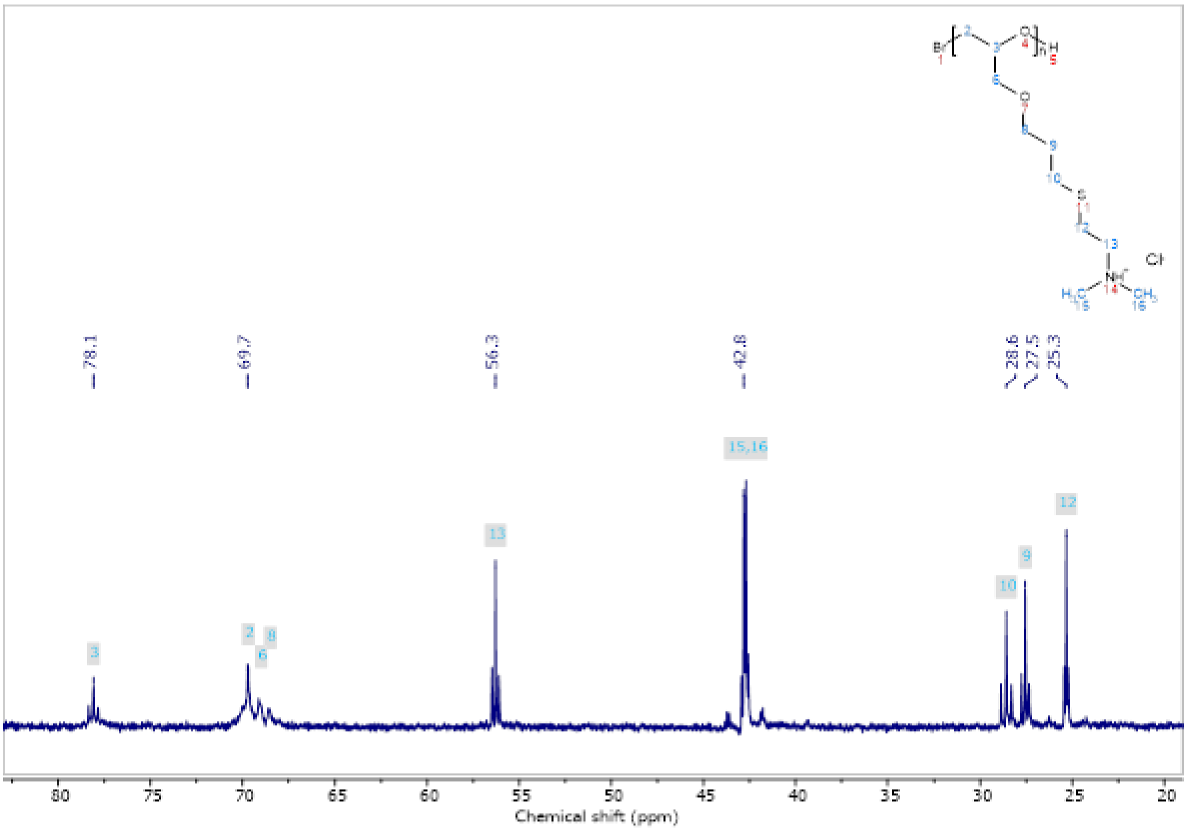

Figure S11. ^{13}C-NMR (176 MHz, D_2O) of polymer **4** (PAGE-$NHMe_2Cl$).

1.3 Summary of molecular characteristics of PAGE and polymers **1-4**

Table S1. Overview of molecular weight of functional polymers **1** – **4**.

Polymer	M_n [kDa]	M_w [kDa]	*Đ*	M_n (Thiol) [g/mol]	Number of r.u.	Degree of functionalization[c]
PAGE	12.1[a]	14.4[a]	1.19	n.a.	125	n.a.
**1**	31.0[b]	36.7[b]	1.19	178.20	125	100%
**2**	37.7[b]	44.6[b]	1.19	241.36	125	100%
**3**	24.1[b]	28.6[b]	1.19	113.60	125	100%
**4**	27.1[b]	32.1[b]	1.19	141.66	125	100%

[a] as obtained from GPC in THF applying PS-standards; [b] as calculated based on the number of r.u.'s and the molecular weight M_n of the respective thiols; [c] determined from ^{1}H NMR; n.a. = not applicable.

2. ITC measurement conditions

Table S2. Summary of used concentrations and measurement conditions in ITC experiments.

Polymer	Polymer Concentration [mM (g/L)]		Protein Concentration [mM (g/L)]		Temperature ϑ [°C]	Ionic Strength [mM]
**1**	0.0273	(1.0)	0.4	(26.5)	25, 30, 37	23
	0.0273	(1.0)	0.4	(26.5)	25	23, 35, 40, 50, 80
**2**	0.01	(0.45)	0.37	(24.4)	25	23
**3**	0.01	(0.29)	0.37	(24.4)	20, 25, 28, 30, 33, 37	23
	0.007	(0.20)	0.4	(26.5)	25	23, 35, 40, 50, 60, 70, 80
**4**	0.01	(0.32)	0.37	(24.4)	20, 25, 28, 30, 33, 37	23
	0.01	(0.32)	0.4	(26.5)	25	23, 35, 40, 50, 60, 70, 80

3. Thermodynamic parameters obtained from ITC measurements

3.1 Representative ITC curves of polymer **1** binding to BSA and thermodynamic parameters

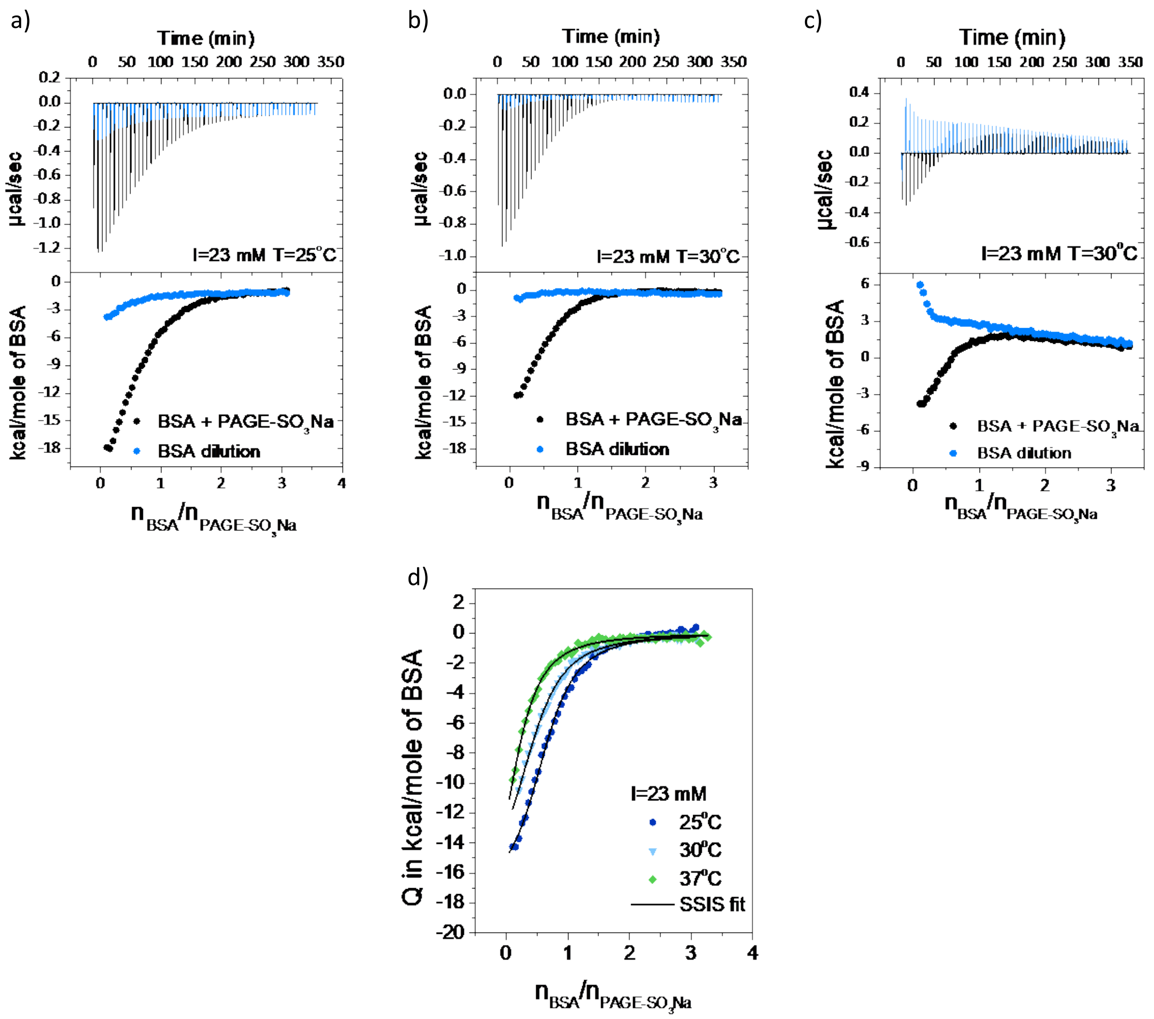

Figure S12. ITC data of the adsorption of BSA onto polymer **1** (PAGE-SO_3Na) (black curves) with the corresponding heats of dilution of BSA (blue curves) at pH=7.4, *I*=23 mM and a) *T* =25°C, b) *T* =30°C, c) *T* =37°C. d) Binding isotherms corrected for the heat of dilution at *T* = 25, 30, 37°C, and 23 mM.

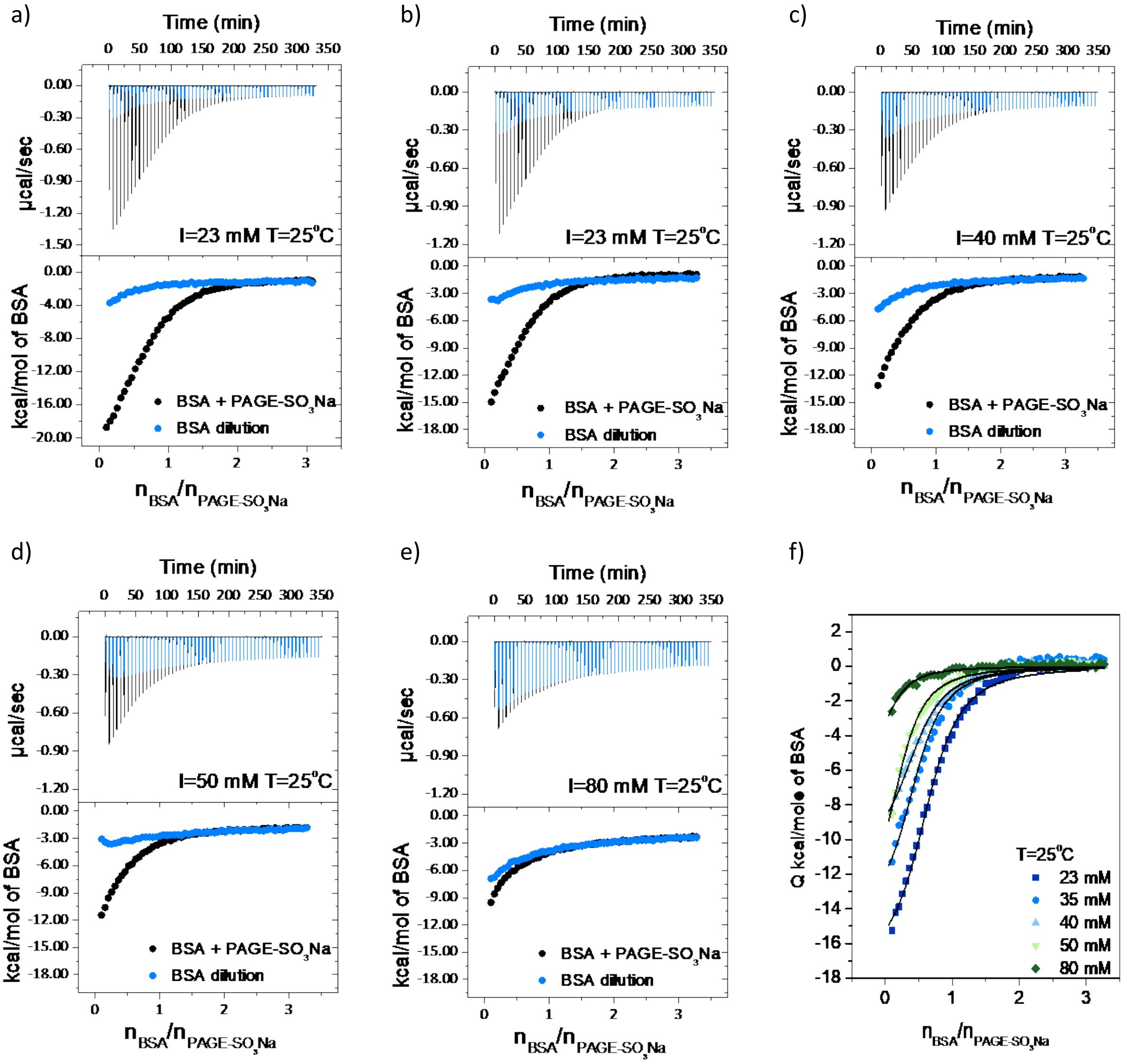

Figure S13. ITC data of adsorption of BSA onto polymer **1** (PAGE-SO$_3$Na) (black curves) with the corresponding heats of dilution of BSA (blue curves) at pH=7.4, T=25°C, and a) I=23 mM, b) I=35 mM, c) I=40 mM, d) I=50 mM, e) I=80mM. d) Binding isotherms corrected for the heat of dilution at T=25°C for all ionic strengths.

Table S3. Thermodynamic parameters of the binding between BSA (c=0.4 mM) and polymer **1** (PAGE-SO$_3$Na) (c=0.03 mM) obtained from fitted binding isotherms.

I [mM]	Temperature ϑ [°C]	N	K_b x 10^{-5} [M^{-1}]	ΔH^{ITC} [kJ/mol]	ΔG_b [kJ/mol]
**23**	25	0.7	2.75 ± 0.45	-74.1 ± 2.4	-31.0 ± 0.4
	30	0.5	2.59 ± 0.22	-68.5 ± 1.0	-31.4 ± 0.3
	37	0.3	1.69 ± 0.06	-81.1 ± 1.1	-31.0 ± 0.1
**35**	25	0.5	2.97 ± 0.35	-61.1 ± 1.9	-31.2 ± 0.3
**40**		0.5	2.47 ± 0.19	-46.3 ± 1.0	-30.8 ± 0.2
**50**		0.3	2.20 ± 0.16	-60.7 ± 1.5	-30.5 ± 0.2
**80**		0.2	1.74 ± 0.21	-25.3 ± 1.2	-29.9 ± 0.3

3.2 Representative ITC curves of polymer **2** with BSA

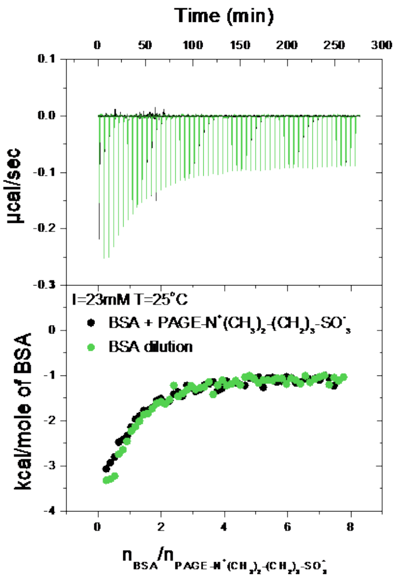

Figure S14. ITC data of adsorption of BSA (c=0.37 mM) onto polymer **2** (PAGE-$N^+(CH_3)_2$ – $(CH_2)_3$ – SO_3^-) (black curves) (c=0.01 mM) with the corresponding heats of dilution (green curves) of BSA at pH=7.4, *I*=23 mM.

3.3 Representative ITC curves of polymer **3** binding to BSA and extracted thermodynamic parameters

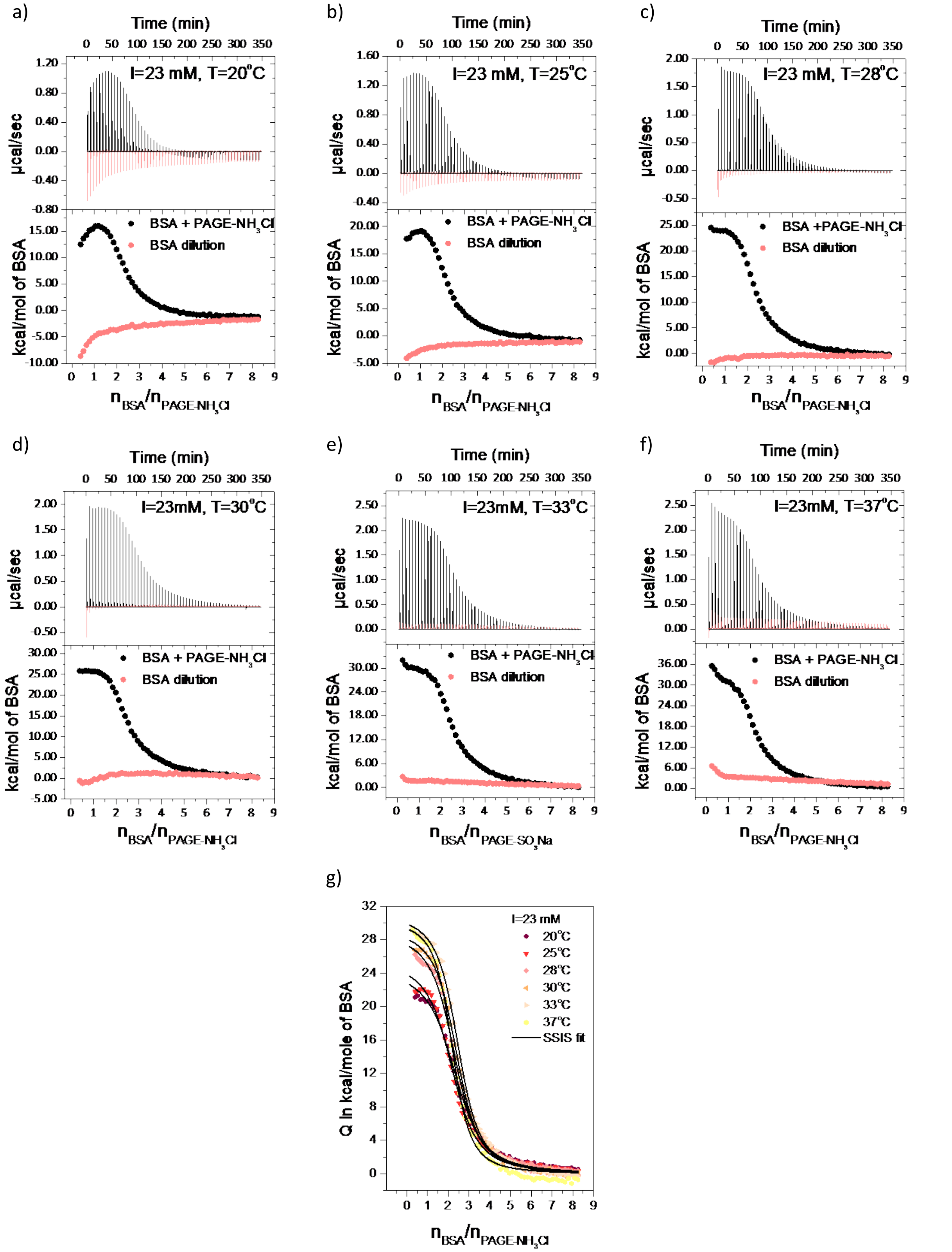

Figure S15. ITC data of adsorption (black curves) of BSA (c=0.37 mM) onto polymer **3** (PAGE-NH_3Cl) (c=0.01 mM) with the corresponding heats of dilution (red curves) of BSA at pH=7.4, *I*=23 mM and a)*T*=20°C b) *T*=25°C, c)

T=28°C, d) T=30°C, e) T=33°C f) T=37°C. g) Binding isotherms corrected for the heat of dilution at T=20, 25, 28, 30, 33, 37°C and I=23 mM.

Table S4. Thermodynamic parameters of binding between BSA (c=0.37 mM) and polymer **3** (PAGE-NH_3Cl) (c=0.01 mM) obtained from fitted isotherms based on a temperature series.

I [mM]	Temperature ϑ [°C]	N	K_b x 10^{-5} [M^{-1}]	ΔH^{ITC} [kJ/mol]	ΔG_b [kJ/mol]
**23**	20	2.5 ± 0.1	5.00 ± 0.37	102.8 ± 1.7	-32.0 ± 0.2
	25	2.3 ± 0.1	5.58 ± 0.42	107.1 ± 1.8	-32.8 ± 0.2
	28	2.3 ± 0.1	6.65 ± 0.39	121.3 ± 1.4	-33.6 ± 0.2
	30	2.4 ± 0.1	7.97 ± 0.37	123.3 ± 1.0	-34.2 ± 0.2
	33	2.5 ± 0.1	8.79 ± 0.46	130.5 ± 1.1	-34.8 ± 0.2
	37	2.2 ± 0.1	11.10 ± 0.87	127.4 ± 1.5	-35.9 ± 0.2

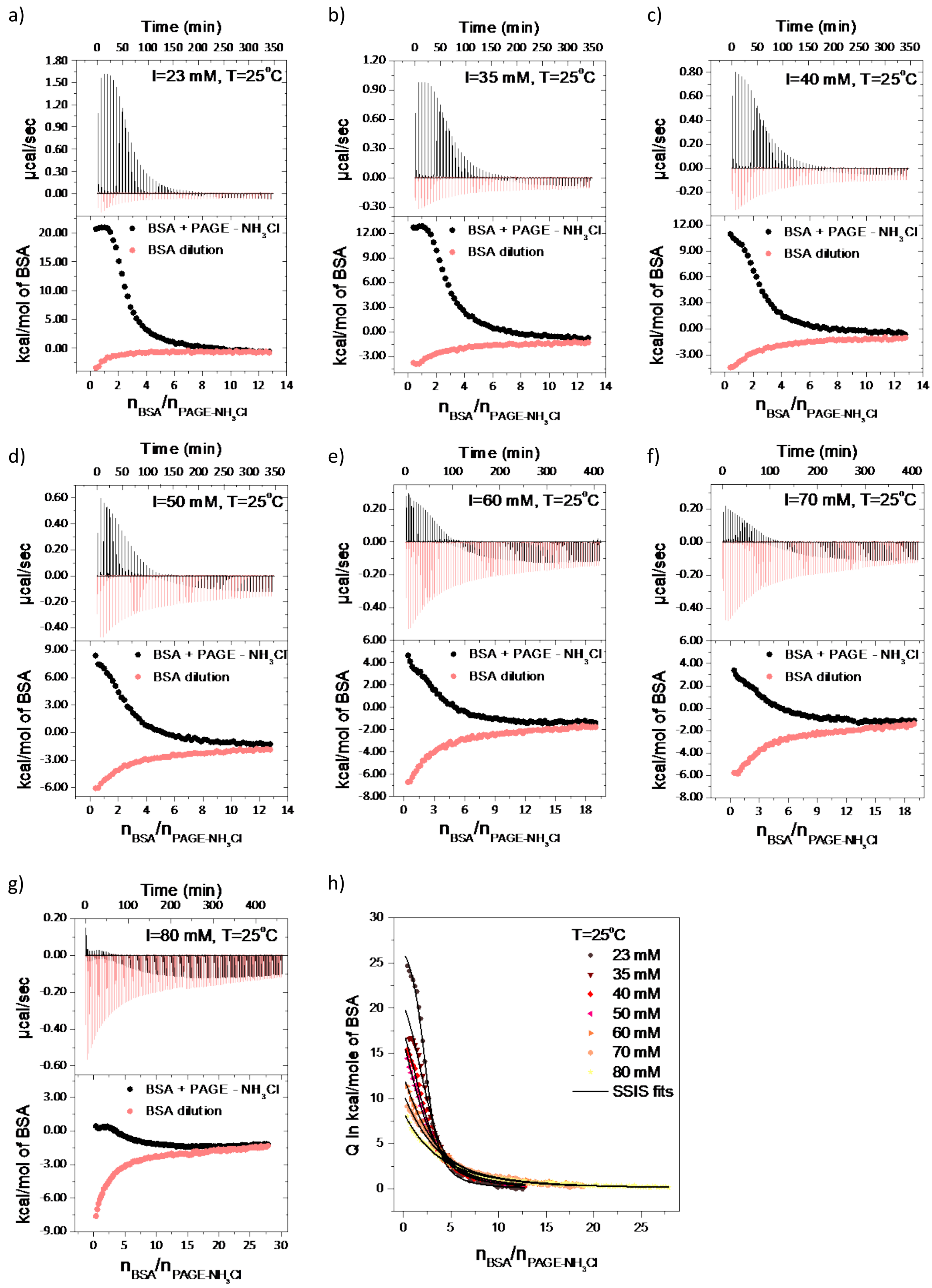

Figure S16. ITC data of the adsorption (black curves) of BSA (c=0.4 mM) onto polymer **3** (PAGE-NH_3Cl) (c=0.007 mM) with the corresponding heats of dilution (red curves) of BSA at pH=7.4, *T*=25°C and a) *I*=23 mM, b) *I*= 35 mM, c) *I*=40 mM, d) *I*=50 mM, e) *I*=60 mM f) *I*=70 mM g) *I*=80 mM. h) Binding isotherms corrected for the heat of dilution at *T*=25°C and *I*=23, 35, 40, 50, 60, 70, 80 mM.

Table S5. Thermodynamic parameters of binding between BSA (c=0.4 mM) and polymer **3** (PAGE-NH_3Cl) (c=0.007 mM) obtained from fitted isotherms based on the ionic strength series.

I **[mM]**	**Temperature ϑ [°C]**	***N***	**K_b x 10^5 [M^{-1}]**	**ΔH^{ITC} [kJ/mol]**	**ΔG_b [kJ/mol]**
**23**	25	2.4	5.35 ± 0.35	120.7 ± 2.0	-32.7 ± 0.2
**35**		2.5	2.06 ± 0.15	107.2 ± 4.1	-30.3 ± 0.2
**40**		2.2	1.54 ± 0.12	100.7 ± 4.4	-29.6 ± 0.2
**50**		2.0	0.96 ± 0.05	114.7 ± 4.5	-28.4 ± 0.2
**60**		2.2	0.54 ± 0.01	112.0 ± 0.9	-27.0 ± 0.1
**70**		2.2	0.42 ± 0.01	109.5 ± 1.1	-26.4 ± 0.1
**80**		2.0	0.33 ± 0.01	109.3 ± 0.9	-25.8 ± 0.1

3.4 Representative ITC curves of polymer **4** binding to BSA and extracted thermodynamic parameters

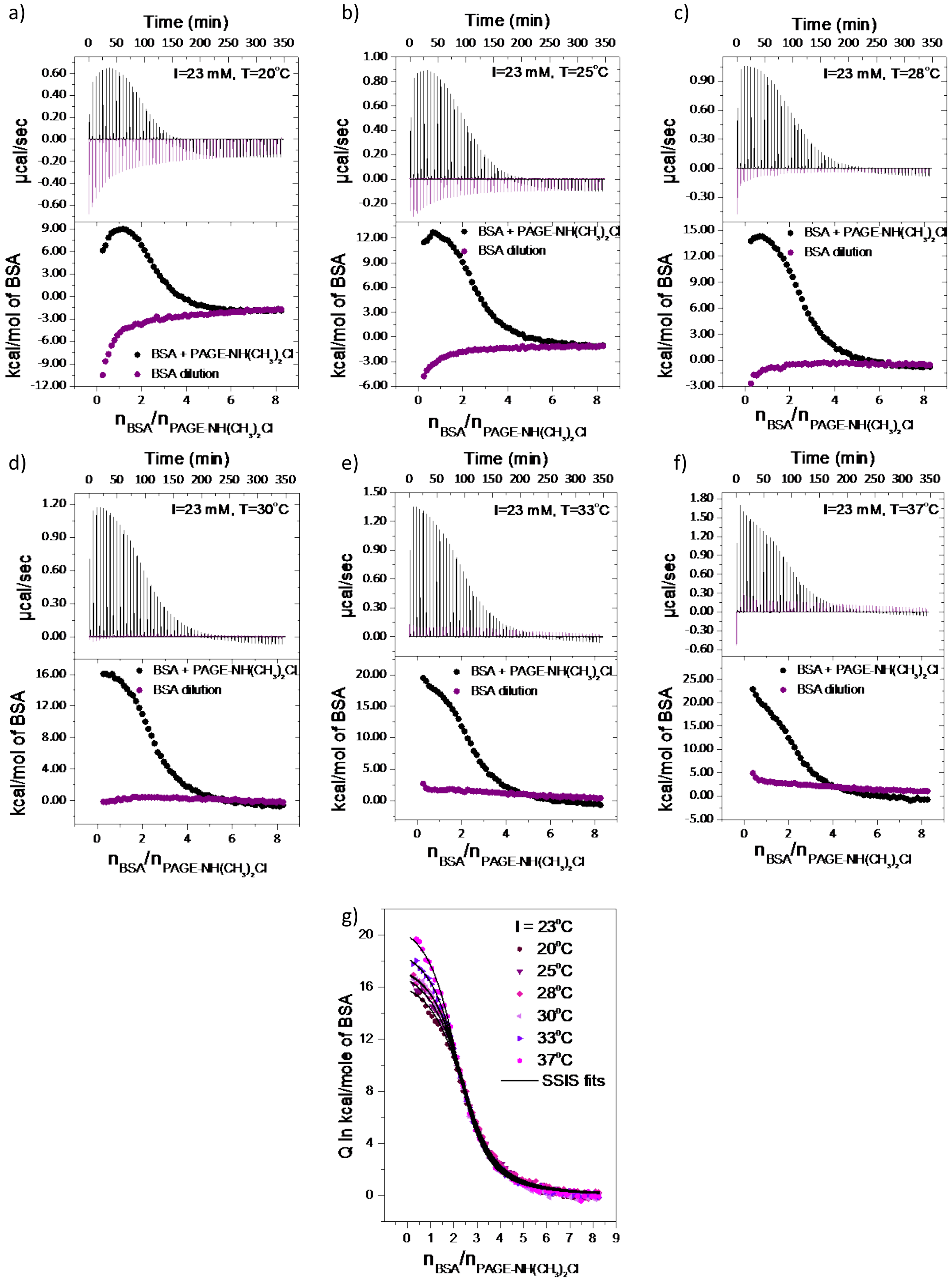

Figure S17. ITC data of adsorption (black curves) of BSA (c=0.37 mM) onto polymer **4** (PAGE-NH(CH_3)$_2$Cl) (c=0.01 mM) with the corresponding heats of dilution (violet curves) of BSA at pH=7.4, *I*=23 mM and a) *T*=20°C

b) *T*=25°C, c) *T*=28°C, d) *T*=30°C, e) *T*=33°C f) *T*=37°C. g) Binding isotherms corrected for the heat of dilution at *T*=20, 25, 28, 30, 33, 37°C and *I*=23 mM.

Table S6. Thermodynamic parameters of binding between BSA (c=0.37 mM) and polymer **4** (PAGE-NH$(CH_3)_2$Cl) (c=0.01 mM) obtained from fitted isotherms based on a temperature series.

I [mM]	Temperature ϑ [°C]	*N*	K_b x 10^5 [M^{-1}]	ΔH^{ITC} [kJ/mol]	ΔG_b [kJ/mol]
**23**	20	2.5	4.85 ± 0.22	71.3 ± 0.7	-31.9 ± 0.2
	25	2.5	4.59 ± 0.16	75.1 ± 0.6	-32.3 ± 0.1
	28	2.5	4.80 ± 0.12	77.0 ± 0.4	-32.7 ± 0.1
	30	2.4	5.12 ± 0.14	78.5 ± 0.5	-33.1 ± 0.1
	33	2.4	5.06 ± 0.14	82.9 ± 0.5	-33.4 ± 0.1
	37	2.3	5.15 ± 0.21	90.5 ± 0.9	-33.9 ± 0.2

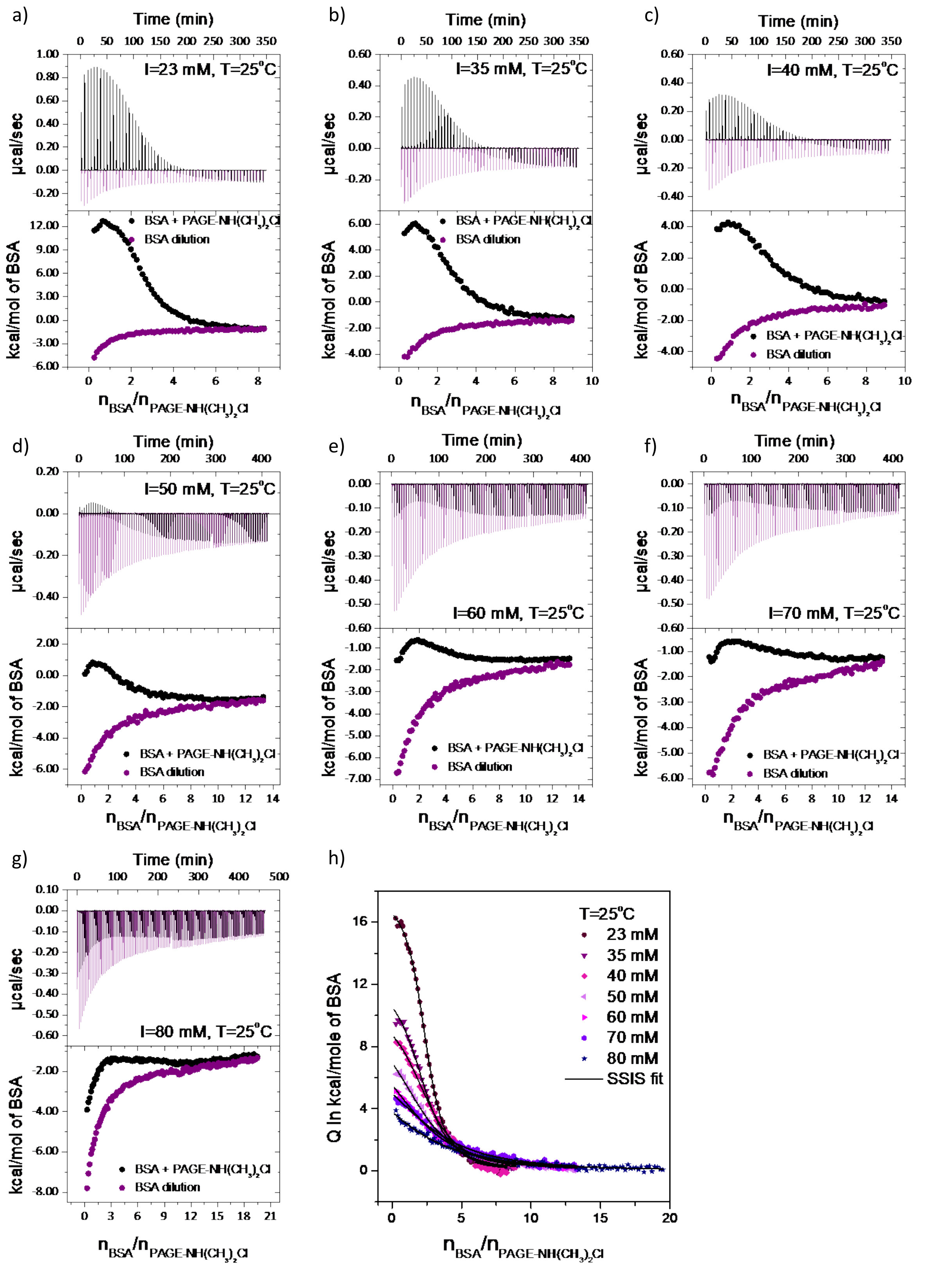

Figure S18. ITC data of the adsorption (black curves) of BSA (c=0.4 mM) onto polymer **4** (PAGE-NH(CH_3)$_2$Cl) (c=0.01 mM) with the corresponding heats of dilution (violet curves) of BSA at pH=7.4, *T*=25°C and a) *I*=23 mM, b) *I*= 35 mM, c) *I*=40 mM, d) *I*=50 mM, e) *I*=60 mM f) *I*=70 mM g) *I*=80 mM. h) Binding isotherms corrected for the heat of dilution at *T*=25°C and *I*=23, 35, 40, 50, 60, 70, 80 mM.

Table S7. Thermodynamic parameters of binding between BSA (c=0.4 mM) and polymer **4** (PAGE-NH(CH_3)$_2$Cl) (c=0.01 mM) obtained from fitted isotherms based on the ionic strength series.

I **[mM]**	**Temperature ϑ [°C]**	***N***	**K_b x 10^{-5} [M^{-1}]**	**ΔH^{ITC} [kJ/mol]**	**ΔG_b [kJ/mol]**
**23**	25	2.5	4.59 ± 0.16	75.1 ± 0.6	-32.3 ± 0.1
**35**		2.5	2.17 ± 0.09	51.8 ± 0.5	-30.4 ± 0.1
**40**		2.8	1.51 ± 0.07	44.8 ± 0.8	-29.5 ± 0.2
**50**		2.6	0.81 ± 0.06	42.7 ± 1.8	-28.0 ± 0.2
**60**		2.6	0.52 ± 0.03	39.4 ± 1.7	-26.9 ± 0.2
**70**		3.1	0.42 ± 0.04	36.4 ± 2.3	-26.4 ± 0.2
**80**		2.8	0.32 ± 0.07	19.5 ± 1.2	-25.7 ± 0.5

a)

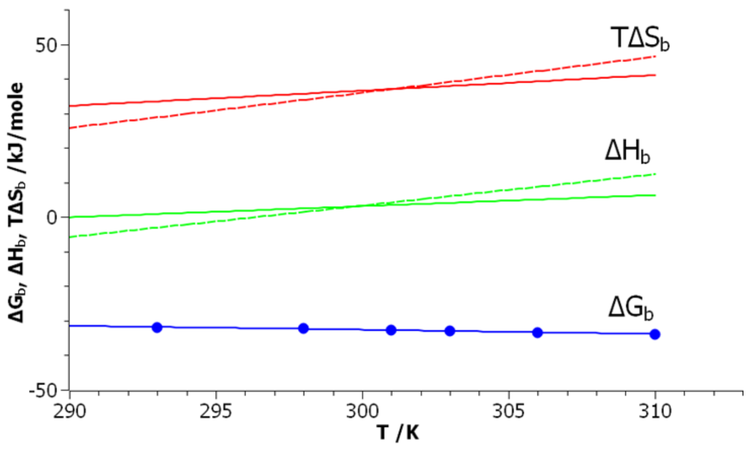

b)

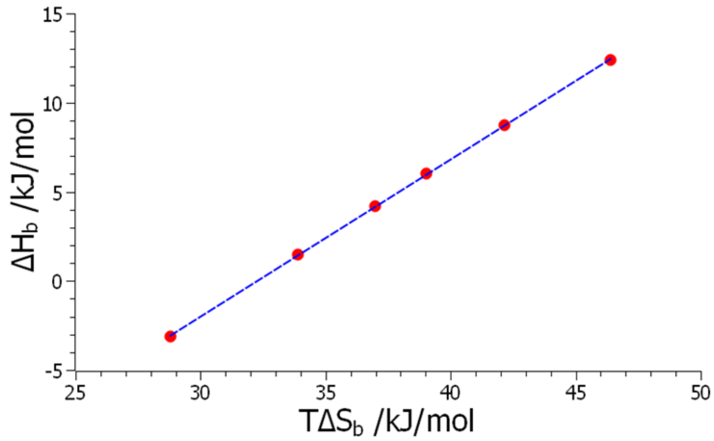

Figure S19. Enthalpy-entropy compensation for the data obtained from polymer **4** (tertiary amine). a) The blue circles denote the measured free energy of binding, whereas the blue solid line gives the respective fit by eq.(14). The solid green and red lines give the enthalpy eq.(15) and the entropy multiplied by T as obtained from eq.(16), respectively. The respective dashed lines denote the enthalpy eq.(4) and the entropy eq.(5) deriving from the fit of eq.(7) to the experimental data. b) The enthalpy ΔH_b (cf. eq.(4)) obtained from the fit of the experimental data according to eq.(7) is plotted against $T\Delta S_b$ (eq.(5)). The slope of the dashed line is 0.88 indicating an incomplete compensation of enthalpy by entropy. See text for further explanation.
